# Integrated genome sizing (IGS) approach for the parallelization of whole genome analysis

**DOI:** 10.1186/s12859-018-2499-1

**Published:** 2018-12-03

**Authors:** Peter Sona, Jong Hui Hong, Sunho Lee, Byong Joon Kim, Woon-Young Hong, Jongcheol Jung, Han-Na Kim, Hyung-Lae Kim, David Christopher, Laurent Herviou, Young Hwan Im, Kwee-Yum Lee, Tae Soon Kim, Jongsun Jung

**Affiliations:** 1 Genome Data Integration Center, Syntekabio Incorporated, Techno-2ro B-512, Yuseong-gu, Daejeon, Republic of Korea 34025; 2 PGM21 (Personalized Genomic Medicine 21), Ewha Womans University Medical Center, 1071, Anyang Cheon-ro, Yangcheon-gu, Seoul, 158-710 Korea; 3 Bioinformatics Solutions, 900 N McCarthy Blvd., Milpitas, CA 95035 USA; 4 Faculty of Medicine, University of Queensland, QLD, Brisbane, 4072 Australia; 5 Department of Clinical Medical Sciences, Seoul National University College of Medicine, 71 Ihwajang-gil, Jongno-gu, Seoul, 03087 South Korea

**Keywords:** Genome sizing, Sequencing, Genome analysis, Statistics, Infrastructure, Storage, Whole genome

## Abstract

**Background:**

The use of whole genome sequence has increased recently with rapid progression of next-generation sequencing (NGS) technologies. However, storing raw sequence reads to perform large-scale genome analysis pose hardware challenges. Despite advancement in genome analytic platforms, efficient approaches remain relevant especially as applied to the human genome. In this study, an Integrated Genome Sizing (IGS) approach is adopted to speed up multiple whole genome analysis in high-performance computing (HPC) environment. The approach splits a genome (GRCh37) into 630 chunks (fragments) wherein multiple chunks can simultaneously be parallelized for sequence analyses across cohorts.

**Results:**

IGS was integrated on Maha-Fs (HPC) system, to provide the parallelization required to analyze 2504 whole genomes. Using a single reference pilot genome, NA12878, we compared the NGS process time between Maha-Fs (NFS SATA hard disk drive) and SGI-UV300 (solid state drive memory). It was observed that SGI-UV300 was faster, having 32.5 mins of process time, while that of the Maha-Fs was 55.2 mins.

**Conclusions:**

The implementation of IGS can leverage the ability of HPC systems to analyze multiple genomes simultaneously. We believe this approach will accelerate research advancement in personalized genomic medicine. Our method is comparable to the fastest methods for sequence alignment.

**Electronic supplementary material:**

The online version of this article (10.1186/s12859-018-2499-1) contains supplementary material, which is available to authorized users.

## Background

The declining cost of generating a DNA sequence is promoting an increase in the uptake of whole genome sequencing (WGS), especially when applied to the human genome. Consequently, the 1000 Genomes Project [1] in the past had integrated the functional spectrum of human genetic variation for approximately 1092 genomes. On the other hand, the genome-wide association studies (GWAS) [2] and the HapMap project [3, 4] have already characterized many sequence variants and their association to match disease phenotypes. Although many sequenced genomes already exist, whole genome and exome sequencing projects [5] have doubled with more data expected to accumulate in the future. A fundamental challenge is the availability of infrastructure and efficient storage designs to aid multiple sequence analyses [6]. Much work is been done in recent years to improve the infrastructure to integrate and process large sequenced data. However, processing the data is computationally resource-intensive, since numerous intermediate analyses require different applications, often having a large size of input data. In this regard, most sequencing studies seek a method that has both higher accuracy and faster speed of performance.

To optimize the computational environment for genetic analysis, we present Integrated Genome Sizing (IGS), a method that splits a full genome sequence into tiny sizable fragments to speed-up genome analysis. The IGS approach is useful in two ways i) It provides leverage on the scalability of high-performance computing (HPC) platforms to improve the NGS processing time through parallel computing, ii) It organizes genome information in a matrix format enabling easy selection of genome portions of interest for analysis. Genome fragments for analysis are chosen in relation to the topic of interest and the total number of fragments used depends on the available computing nodes. Two fundamental aspects - genome sizing and system performance – were considered in IGS. Firstly, the genome sizing uses a concept of storing and localizing sequence data, which seeks to reduce the size of input data for improving the system performance. Each genome is split into 630 chromosome fragments called chunks (Additional file 1: Table S1). A chunk is a nucleotide sequence with an average size of approximately 5 MB. The principle behind choosing the appropriate chunk size was based on the expectation to extend the IGS database with more samples in the future. Considering the size of a single genome, the assumption to assemble about 10,000 full genome samples in a matrix format, ignited the cognition that the estimated minimum size for each chuck should be 5 MB, since 5 MB * 10,000 samples are approximately 50 GB. Therefore, with a 64 GB memory, we can assemble over 10 thousand samples in a matrix. In addition, if the data is further compressed to binary format, more memory will be saved. The concept is feasible as it will allow more data expansion over a period of time. The current chunk data represents the Human assembly version GRCh37. We have equally fragmented the genome version GRCh38. Though some very slight changes were observed at some chromosome regions, the complete comparison for both versions will be made available in the future upgraded version of IGS.

Storage is provided by Maha-Fs (ETRI, Korea) [7], a system build on the Remote Direct Storage Management [8] which enables an effective processing of files and storage on a client server. Maha-Fs can process 200 jobs simultaneously consisting of 1600 cores of CPUs, 8 cores, and 64 GB memory per node (default setting), 1.4 petabytes of hard drives, and 10 GB of Ethernet. The metadata server of Maha-Fs supports multiple disks types including solid-state drives and hard disk drives. About 2504 whole genomes are currently hosted on Maha-Fs. Nevertheless, such a dataset is too large to be processed by sheer computational power alone, and it is practically difficult for conducting association studies across samples using a single computer. Analyzing a genome sequence implies that one needs to trace locations of over 3 billion nucleotide bases from raw sequence reads. This results in significant bottlenecks due to a limited and inefficient storage management system. A systematic storage approach should be able to organize data for easy extraction and subsequently improve data communication across different programs during data processing. In a typical genome fragmentation report, a method called MegaSeq [9] was designed to harness the size and memory of the Cray XE6 supercomputer, which greatly sped up the variant calling time for 240 genomes through parallel analysis. When implementing the MegSeq workflow, each genome was split into 2400 units to take advantage of the Cray XE6 system.

**Fig. 1:**
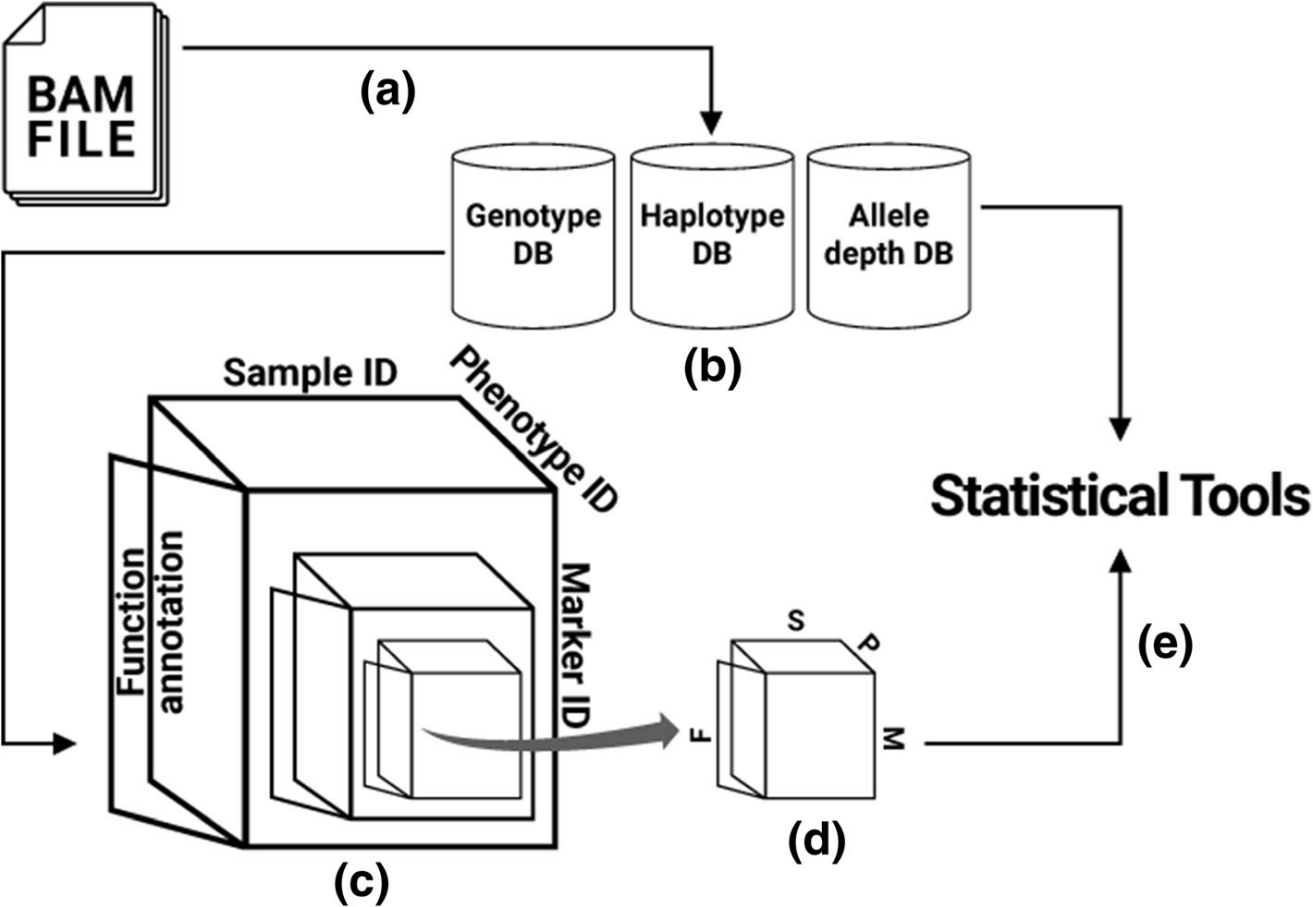
Basic communication and data processing in IGS. A BAM file (**a**) used for generating three major databases (**b**). The data is organically arranged and cloned into four-dimensional (4-D) information (Phenotype variable ID, Marker ID, Sample ID, and Function annotation) as shown in panel (**c**). In each request, IGS extracts 4-D data. All extracted information is a sub-clone (**d**), and the data is subjected to an in-house statistical tool, IGscan (**e**), which provides statistical analyses

In the past, genome analysis relied on publicly available platforms that integrated sequence data stored across several biological databases [10–12]. Each genome sequence is presented using different data formats and structures, and each distinct data type provides a unique, partly independent and complementary view of the whole genome [13]. The ClinVar database, for instance, stores relationships among sequence variation and human phenotypes [14], dbSNP archives genetic variations [15], and the Human Gene Mutation database collates sequence variation responsible for inherited diseases [16–19]. When segments of the whole genome are stored in separate locations, indexing and manipulating data can be challenging especially when dealing with a complex project such as GWAS experiments. However, in IGS, segments of the full genome are systematically organized in a relational database fashion where IDs (keys) are assigned for efficient data indexing (Fig. 1c)**.** The IDs allows mapping of related data sets. In this perspective, three distinct IDs were assigned based on the data content including; (1) Marker ID with Chunk ID, (2) sample ID and (3) Phenotype ID**.** The Biomarker/chunk ID denotes a specific bounded region representing an interval of loci in a given chromosome region. Sample IDs are specific identifiers for every sample stored in the database. The IGS sample IDs are automatically updated when new samples are deposited. On the other hand, the phenotype ID represents the phenotypic information for each sample used to index specified marker(SNV) with Chunk ID. This matrix design provides flexibility and benefits to statistical tools for indexing precise information of a queried region of interest. Thus, effective data communication is ensured across all datasets within the system. As a backend package, we designed and adapted an Integrated Genome Scanning (IGscan) package for statistical analysis. It should be noted that, based on IGS setup, researchers, groups, and institutions can easily design tools or customized existing packages to mine data stored herein. The default setting of IGscan algorithm employs; ‘mkey’, ‘skey’ and ‘pkey’ keys, which denote the IDs for Marker with Chunk, samples, and phenotype respectively.

In the next section, we have provided a detailed description of the storage and data structure of IGS with an example of its applications. In the Results section, we have outlined the scalability of IGS as a strategy for comparing the performances of Maha-Fs and SGI-UV300 (HP, USA California) systems using a reference genome, NA12878. A brief outline of an exemplary usage of the IGScan QC (analytic) module, a customized statistical toolbox, is also provided.

## Methods

### Genome sizing approach

**Fig. 2:**
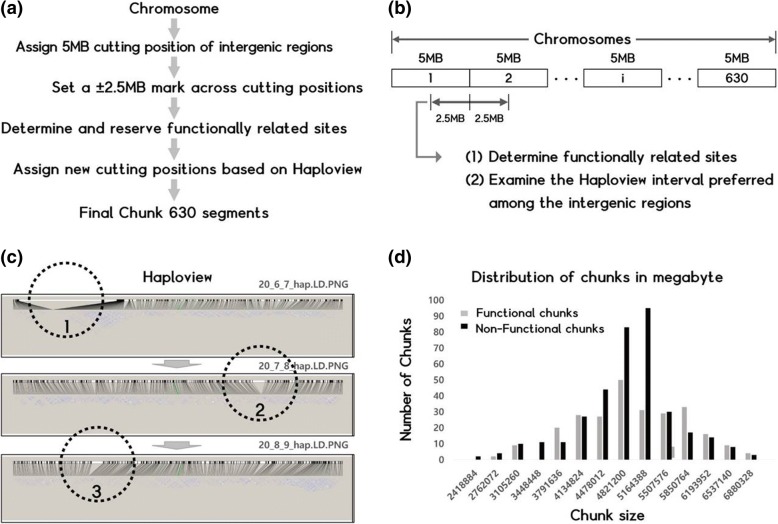
Schematic representation of genome chunking workflow. **a** A step-by-step procedure involved in creating 630 chunks from a genome. **b** Generation of chunks by setting cut points, and practical steps involved in creating chunks from a given chromosome length. The entire genome is divided into 5 Mb segments (*n* = 630) by making virtual cuts. Next, a 2.5 Mb distance is added to both ends of the initial cut point to determine the presence of functional sites and to allow Haploview interval analysis among intergenic regions. **c** Haploview analysis: The three distinctive regions, marked 1, 2, and 3 are the new cut points of a chunk selected by Haploview analysis to identify the relationships among the selected SNPs. We recalculated the length of each cut point to include related biological information to obtain informative chunks (as denoted by 20_6_7_hap.LD.PNG, 20_7_8_hap.LD.PNG, and 20_8_9_hap.LD.PNG, respectively). These regions represent a precise sequence information ranging from 4 to 6 Mb in length, which could be information related to CNV or genes. **d** Distribution of chunks. The graph illustrates the distribution of functionally related chunks along with functionally unrelated chunks and the classification of chunks based on their respective numbers of markers

As shown in Fig. 1a, the IUPAC binary alignment map (BAM) sequence reads for 2504 genomes [20] generated by BWA-MEM [21] were extracted. Each genome was split by chromosome irrespective of size, by setting up initial points for a virtual cut. An intergenic interval included a nucleotide sequence of specific length of 5 Mb, and a 2.5 Mb distance was added to both ends of the initial cut points (Fig. 2a). From here, we examined the fragments by recalculating the cut points based on biologically relevant information (Fig. 2b). Next, the Haploview analysis (Fig. 2c) was performed by only using SNP information, with the following criteria for marker selection: (1) Minor allele frequency (MAF) > 0.05; (2) Call rate > 0.75 and (3) Hardy-Weinberg *P*-value < 0.001. After identifying the relationships between the selected SNPs, we set the midpoint of the region with the longest distance between the markers as a new virtual cut point. Finally, we identified annotated information relevant to biological functions, such as (1) Cytoband region related to diseases and certain functions from the CytoOneArray Phalanx database (Additional file 2: Table S2), (2) Copy number variation (CNV) related to rare diseases obtained from two sources; CNV in Clinvar database (Additional file 3: Table S3) and CNVD:- copy number variation in disease database (Additional file 4: Table S4), (3) NCBI Map Viewer for mapping phenotype information (Additional file 5: Table S5) and finally, (4) Genetic information. The information was synthesized to set and rearrange the cut points such that the average distances between them fell in the range 4 MB to 6 MB. The Cytoband regions within lengthy chunks were divided and the new cut points were carefully made to avoid affecting genes. In this sense, a chunk is a specific chromosome portion measuring about 5 Mb of the base sequence that may contain known functional regions of a genome. It can also be viewed as a fraction of a given chromosome length out of the total size of the genome. The configuration of a chunk was necessary to solve the limitation of computational resource that arises when constructing and using the database. In addition, the configuration of the database into chunks preserved the information about biological function relevant to the genome.

Of the 630 chunks, 82% fell between 4 Mb and 6 Mb in length (Fig. 2d), and about 43% had known biological functions as shown chunk list (Additional file 1: Table S1). For example, the human leukocyte antigen (HLA) system is the major histocompatibility complex (MHC), involved in recognition of exogenous proteins or peptides by the immune system. The HLA varies from person to person and is related to certain diseases [22, 23] and drug reactivity [24, 25], as well as immunity. The whole HLA region is located on the short arm (6p21.2–21.3) of chromosome 6. In IGS, the region (6_29,678,325-6_35,156,630) is mapped to the chunk ID ‘6_7_220’ (Table 1). It becomes substantially more efficient to handle the HLA allele information by selecting the specific chunk from its location, bypassing the querying process of the entire database. Moreover, a database of selected chunks can easily be used for disease association amongst them.

**Table 1:** Example of chunk distribution of chromosome 6 of the reference genome

Chunk ID	Chrs	Start	End	Related Function
6_7_220	6	29,678,325	35,156,630	HLA region
6_8_221	6	35,156,631	40,140,014	–
6_9_222	6	40,140,015	46,461,804	Microvascular_complications_of_diabetes_1
6_10_223	6	46,461,805	49,686,975	–
6_11_224	6	49,686,976	55,283,232	–
6_12_225	6	55,283,233	60,365,606	–
6_13_226	6	60,365,607	66,419,118	Epilepsy/Dysle23ia/EYES SHUT /DROSOPHIL
6_14_227	6	66,419,119	67,721,229	–
6_15_228	6	67,721,230	73,114,845	–
6_16_229	6	73,114,846	77,870,236	–

The above partial table depicts detailed information for 10 selected chunks in chromosome 6. The full list is provided in the Additional file 1:Table S1, consisting of six columns and 630 rows. The first column represents the chunk ID and is composed of three subentries separated by an underscore ‘_’. The first entry stands for a chromosome from which the chunk was obtained. The second entry represents a chunk number within that chromosome, and the third entry is a global chunk number within an entire whole genome. The second column stands for a chromosome type. The third and fourth columns are the chromosome Start and End positions respectively, while the fifth column is the curated function-related data of the designated chunk. If its functional role is not determined, a minus sign ‘-’ is assigned to this field. Finally, the sixth column (not shown) is the specific functional chunk region. Each complete row represents a single independent chunk.

## Structure of IGS and data storage

**Fig. 3:**
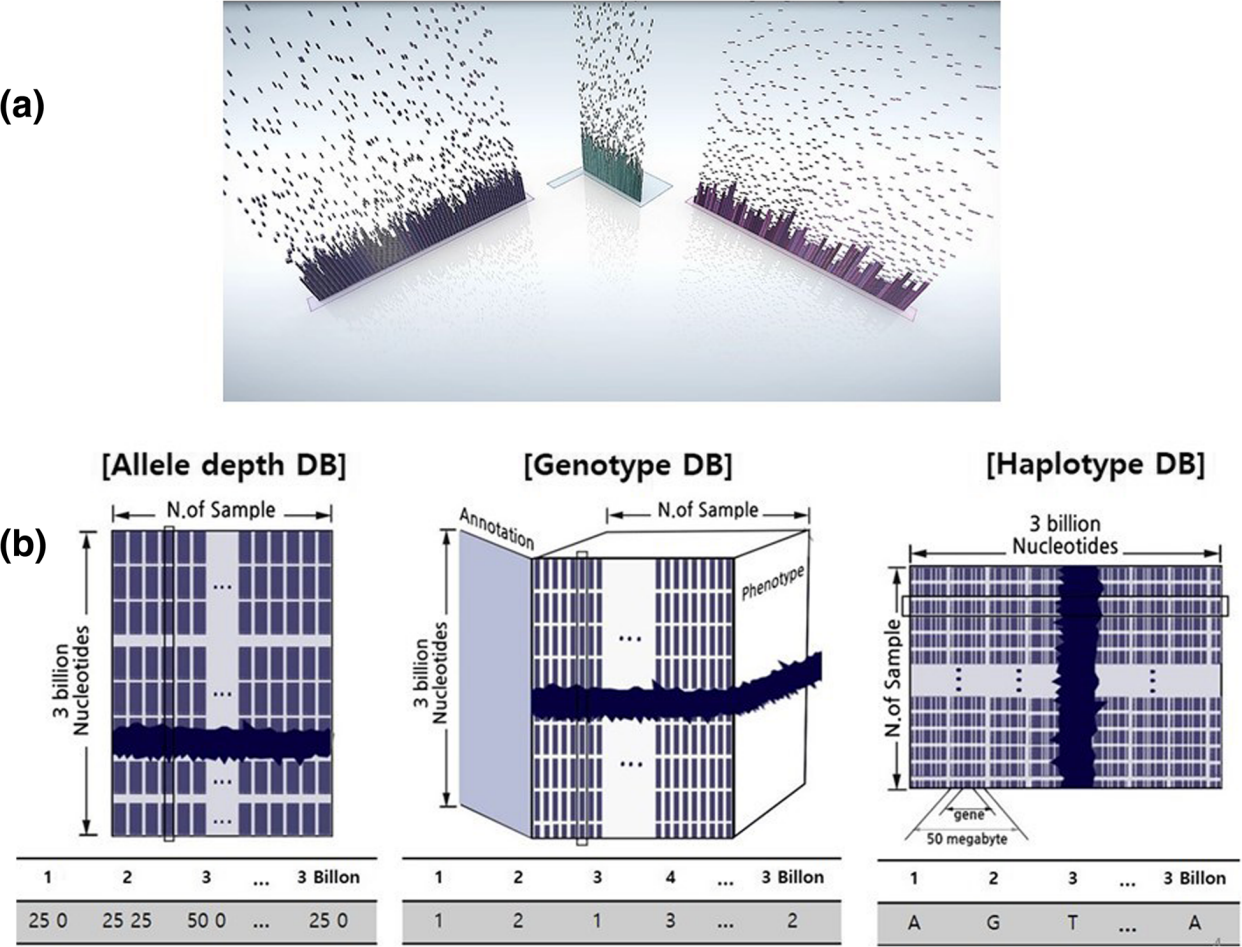
Three mosaic structures representing the organization of chunks. **a** A data point of allele depth, genotype, or haplotype. Each dot designates a single chunk entity. Repeated addition of chunks yields (**b**) a matrix of three databases

Figure 3a shows dots representing individual chunks. To organize and store sequenced data pertaining to each chunk, a genotyping algorithm ADIScan 2 [26] was used to extract genotype information. The same process can be achieved using known genotyping software. In IGS, the variant calling algorithm ADIScan 1 [27] and the haplotyping algorithm HLAscan [28] were used to extract strings of genotype, allele depth, and IUPAC haplotype from each dot respectively. This process was repeated continuously by adding a dot sequentially to build three distinctive databases that are coordinated in a fully related manner, similar to a relational database (Fig. 3b). The first is the ‘Allele Depth’ database which comprises information about allele depth and quality information for each sample at a specific chromosome position. This information can be used to predict the phenotype of selected samples in relation to a variety of disease episodes by calculating the reliability and rarity of variation at a given position using statistical tools. The second is the ‘Haplotype’ database, which consists of IUPAC codes and the causes of various phenotypes, such as eye and hair color, personal constitutions, ethnic characteristics, and diseases, which can be predicted using haplotype information. The last is the ‘Genotype’ database which hosts genetic trait information for each location of a chromosome, the function of each gene within this location, the phenotypic information, and the relationship among the samples. Based on the Genotype database, statistical analysis can be performed using IGscan tool (IGScan- freely available on request for non-commercial purposes) for thousands of control and disease cases of selected sequence regions and which can also generates input file formats of popular applications such as Fbat [29], Plink [30], Merlin [31, 32], Linkage [33], Phase [34], and Structure [35]. This database is also compatible with most integrated genetic database applications.

Currently, the IGS supports 2504 individual genomes from 26 ethnic populations. Researchers can take advantage of IGS to perform a wide range of genetic analysis focusing on any chosen genome region of interest. Furthermore, patient stratification for fast-track drug discovery can be performed on a selected disease, involving all samples within the database.

### Application of IGS

The IGS data is organized in four-dimensional (4-D) indexing matrix (Fig. 1c). The design allows rapid data retrieval from specific sequence regions across multiple samples using statistical tools (Fig. 1e). This was illustrated by implementing an in-house statistical tool designed with a number of APIs for accessing IGS databases and generate statistical results. Each API module produces different statistical output based on its parameters and the type of results expected. Being a multi-functional algorithm, IGScan can be implemented in several ways including (1) building newly integrated databases; (2) statistical analysis that can determine the quality of a marker according to samples in a constituent database; and (3) generation of standard input file formats for the previously cited standard software packages widely employed in bioinformatics and so on. All the three IDs of IGS for data mapping are organized in a three-dimensional (3-D) correlation matrix: the x-axis refers to a genotyped marker (mkeys), the y-axis refers to a phenotype (pkeys), and the z-axis represents a sample (skeys). Therefore, given the coordinates (x, y, z) of a defined genome region (“mkeys”, “pkeys”, and “skeys”), including functional annotation information (or even their input files), IGScan can recognize 3-D relationships among the samples, phenotypes, and markers of genotypes and utilize them in calculating the statistical relationships of their 3-D correlation matrices, as well as generating annotations. The application of a particular module depends on research interest. Another important feature of IGS is accounting for the properties of a matrix as an integrated whole and generating basic statistics, such as quality control of genotype, phenotype, and functional annotation. For instance, given a pathway involved in a drug-targeting mechanism, one can extract information of genes related to a target pathway and of their genotypes, along with examining the statistical significance of their association. Taking advantage of the system’s ability to rapidly access information of the relevant phenotype, genotype, and function annotation, one can extract the data simultaneously with a single API command.

## Results

The IGS is a whole-genome sizing approach, which incorporates 630 small datasets and provides several advantages: (1) reduction of data size and convenient storage; (2) specific localization of data; (3) direct access to target data; and (4) fast NGS processing time with parallelization of ~ 600 distributed jobs.

### Comparison of NGS processing speed across HPC systems

To perform computation and store data for NGS processing with HPC system, it requires not only specialized algorithms but also appropriate hardware. Two main hardware-related problems that must be addressed are - the time-cost of data analysis in processing steps; and the security and protection against failure of hardware storage [36]. To resolve the former, we conducted a pilot test using our NGS pipeline to evaluate the processing speed for analyzing a reference genome, NA12878 [37], using two different infrastructures - Maha-Fs (ETRI, Korea) and SGI-UV300 (HP, USA California) - both with 4 or 16 CPU cores and 12 or 64 GB of memory per node. When a FASTQ file was divided into 630 chunks, processing of the whole-genome sequence took approximately 55.2 mins using Maha-Fs and 32.2 mins using SGI-UV300.

Table 2 shows the speed results of ten processing steps (mapping and recalibration) in both hardwares. The results show that the SGI-UV-300 was approximately twice as fast as Maha-Fs. The high performance of SGI-UV300 is related to its solid-state drive (SSD) storage design, wherein information is stored in a microchip memory. This yielded an improved performance when compared to the network file system (NFS) and the distributed parallel architecture-based SATA hard disk drive (HDD) of Maha-Fs. In particular, the splitting process (step 1) of SGI-UV300, regardless of the number of CPU cores, was five times faster than that of Maha-Fs. The result implies a five-fold reduction in the I/O dependency of SGI-UV300 simply because it only involves splitting one full genome into tiny chunks (630). However, the results of BWA-MEM in step 3, Picard-fix mate information in step 4, and GATK-related issues in steps 6 to 10 revealed that the performance of both systems was dependent on the number of CPU cores and size of memory. Maha-Fs, which uses a distributed parallel computing in multi-core dependency steps, exhibited slightly better performance, implying that the issue of multi-core traffic is less critical in Maha-Fs than in SGI- UV300 system. In addition, we conducted an experiment to determine the optimal memory usage and other parameters for analyzing one chunk using nine pipeline processes except for the split step (1.split). Here, we employed two resource environments (local disk and Maha). We set up three different parameters for both systems. Para 1: 4cores/20GB memory, para 2: 8cores/30GB memory and para 3: 8cores/64GB memory respectively. The para 3 was the default setting and this parameter was common on both systems. However, the para 1 and para 2 were unique for the local disk and maha environment respectively. We then created four experimental cases, where the local disk was entitled to para 1 (case 1) and para 3 (case 2), while maha was set to para 2 (case 3) and para 3 (case 4) respectively. While cases 2 & 4 parameters were set at default, cases 1 & 3 were used for evaluation. The maximum network used was 1GB (Max 120 MB receive/send) in all cases. For cases 1 & 3 analyses, it was confirmed that only an average of 12GB memory was utilized. Addition of more memory produced no gain. The optimal processing parameters recorded were 4cores/12GB memory across all steps even though the amount of memory available was large. For instance, the read trimmer (sickle – step 2) depicts 4GB memory used on the local disk environment, while, only 1GB memory was utilized by maha (Additional file 6: Figure S1).

**Table 2:** Performance comparison of Maha-Fs and SGI-UV300

Method	Process Steps	Maha-Fs		SGI-UV300	
Core:Memory		4:12	16:64	4:12	16:64
mapping	1. split	35.1	37.7	7.1	7.1
	2. sickle	1.2	0.4	2.2	0.2
	3. BWA-MEM	9.4	2.5	7.6	3.1
	4. Picard-Fix Mate Information	2.6	1.9	4.3	2.7
recalibration	5. Picard-Mark Duplicates	1.5	1.1	7.3	2.7
	6. GATK-RealignerTarget Creator	2.9	1.8	4.2	2.7
	7. GATK-Indel Re-aligner	1.6	1.1	2.7	1.7
	8. GATK-Base Re-calibrator	2.1	1.0	5.5	1.1
	9. GATK-Print Reads	2.9	2.2	7.4	3.2
	10. GATK-Haplotype Caller	8.0	5.6	10.8	8.1
Time	Total process time (min)	79.8	55.2	59.0	32.5

Currently, the field programmable gate array (FPGA) is the fastest microchip in whole-genome NGS data processing. A previous study showed that implementing FPGA in the Dragen pipeline took approximately 40 min for WGS alignment and variant calling [38] using the same NA12878 genome. In view of our results, we can conclude that, depending on the application and scalability, SGI-UV300 (with SSD, multiple cores, and memory), Dragen (with advanced FPGA-chip), and Maha-Fs (with distributed parallel computing) were reasonably comparable in terms of high-performance computing in NGS processing.

The varying levels of speed between Maha-Fs and SGI-UV300 HPC systems using a reference genome in the same pipeline are shown in Table 2. Maha-Fs consists of a SATA hard disk drive (HDD) storage, while SGI-UV300 has a solid state drive (SSD) memory which runs faster than the former. In the results, SGI-UV300 completed the job in 32.2 mins and Maha-Fs in 55.2 mins. The first two columns stand for methods and steps used in the pipeline process, whereas the last two are the performance statistics of Maha-Fs and SGI-UV300, respectively.

By default, it takes an average of 7 h to consolidate one independent chunk after separation of a genome sequence into 630 units. Consolidating 630 chunks of 2504 genomes using 1 Maha-Fs unit of hardware took 3.5 days in total, but the theoretical estimated time was 21 h ([630 chunks * 7 h] / 200 jobs). Both of the average times spent in consolidating all chunks and a single chunk (7 h) were nearly four times longer than the estimated times due to the heavy load on storage I/O, and such issues are commonly encountered in whole-genome analyses. The huge I/O cost probably occurred due to use of 1GB (gigabyte) network card mounted at the time. This was concordant with an earlier I/O stress experiment conducted to show the stress levels when the load (job) size increases progressively. At a certain point of job count, no I/O issues were recorded. However above this limit, load increment causes disk delay due to simultaneous reading and writing processes eventually taking up more time.

### Example of statistical test

Statistical tools can be deployed or integrated with the IGS to take advantage of its data pattern. Using IGscan, we performed a statistical test to determine the quality of information on the BRCA1 gene across 2504 samples (Additional file 7: Table S6). Here, an example of IGScan command **$ ‘IGscan –a QC mkey_file [input 1] skey_file [input 2] –r /DB/path……/’,** would provide statistical analysis of a selected region of interest**.** Where **–a** stands for analysis**, QC** is the API for mining quality information a chosen genome region of interest. The **mkey_file** denote**s** the biomarker input file **(input 1)** and **skey_file** represent the sample input file **(input 2)** respectively**.** Lastly, **−r/DB/path…/ is** the database path. First, all the sequence loci along the selected BRCA1 gene portion are extracted. The said sequence corresponds to a specific chunk within the IGS database. Secondly, using a Python script, the chunk representing the region is automatically indexed **[input 1].** The sample input file **[input 2]** is created by collecting all sample IDs for cross analysis. Running the **QC** analysis on a single chunk took 15 min (Table 3). Meanwhile, it took roughly 60 min to analyze 630 chunks across 2504 samples in 4 cycles (200 nodes per cycle). An example of generated QC results (data not shown) is found in the Additional file 8: Table S7. The QC module can be replaced by other APIs to generate different statistics. IGscan is capable of handling multiple logistic or linear regressions, and all types of chi-squares (including covariance operations) against a single genotype, multiple genotypes, a single phenotype, or multiple phenotypes. This tool will be made freely available to the research community in the future to facilitate genome studies.

**Table 3:** IGscan QC analysis Vs number of chunk use

Module	No. samples	No. chunks	Time (mins)
QC	2504	1	15
QC	2504	630	60

The table shows the variation of time for analyzing a varying number of chunks. The QC operation took 60 min for full genome (630 chunks) as opposed to 15 when a single chunk is used.

## Discussion

Precision medicine uses the personal medical information to diagnose and tailor medications and management plans for treating diseases and improving health [39]. This approach is expected to enable the medical community to select the best clinical practice for individual patients based on their genetic information. As the use of NGS grows, the scope of its application is also expanding, especially in the areas of clinical diagnosis and validation [40]. Converting 1D nucleotide sequence to 2D image with genetic variants and phenotypes is standardized and differentiated from one another for deep learning analysis with a convolutional neural network (CNN). In this regard, IGS is very useful as any genomic region of interest can be easily selected and filtered by statistics, pathway-related genes, targeted genes, phenotype, sex, ethnic group and diploid-based variants [41–44]. As personalized healthcare relies on accurate analytics to guide decision-making, it creates a high demand for more practical ways of handling WGS. Nevertheless, the evolution of WGS has been partially hindered by the challenges to store and manipulate large nucleotide strings. The most commonly applied approach to date has been the use of a central system to integrate data residing in remote resources [45]. Unfortunately, this method is not adequate for handling multiple cohort analysis. Another challenge is related to the high heterogeneity of data in such databases, which could compromise the quality of data [46].

The goal of IGS is to allow efficient management, processing, and analysis WGS of multiple subjects in a simultaneous manner. When a long genome sequence is organized into small units within the same schema, the overall communication speed required for data extraction becomes significantly faster, which suits the analysis of larger datasets. In terms of HPC of NGS processing, our results demonstrated that the Maha-Fs, SGI-UV300, and advanced FPGA-chip were all comparable, only differing on their application and scalability. Contrasting the IGS with a similar approach by Puckelwartz et al., the Cray XE6 supercomputing system was adapted for whole-genome parallelization. This system comprises of 726 nodes with 34 GB and 24 cores per node. Similar to the work reported here, the study implemented a concept of splitting the whole genome into smaller pieces. Computational speed was doubled, and efficient parallelization was achieved. Nevertheless the method, unlike IGS, focus on variant calling rather than multiple genome integration. Furthermore, the number of whole genomes used (240) was small relative to that used in the current study.

Several requirements exist to implement IGS - a user to be trained in how to operate the system, manipulate data, and interpret results, as well as a storage facility to be available in hosting data for more than 1000 genomes. The latter requirement could be a major block for private researchers and organizations with less robust infrastructure. Nevertheless, full functionality of IGS is sure to benefit larger organizations capable of providing the required storage, since IGS can provide accurate and detailed information of the whole genome. Moreover, it will allow predicting numerous phenomena in genome association studies. For example, genome integration may provide predictions regarding which illnesses a patient may experience in the future, thus ensuring better management strategies to prevent diseases.

## Conclusion

As genome-sequencing techniques currently guide precision medicine, demands for reliable and rapid NGS methods are gaining momentum in medical professions and manufacturers of therapeutic agents. Although we are witnessing an ongoing development of the infrastructural aspect of the method, efficient manipulation of NGS data for the whole human genome still remains a challenge. IGS enables convenient analyses of whole sequences across multiple samples, markedly improving the computation time. We believe that IGS could open new avenues for rapid and multifunctional genome sequencing that can deal with large volume of data.

## Additional files

Additional file 1: **Table S1.** Chunk Distribution Across The Reference Genome (Human Assembly Version GRCh37). File consists of all annotated chunk regions mapped to some specific function. The number of chunks derived from each chromosome is represented. (XLSX 46 kb)

Additional file 2: **Table S2.** List of Disease Related Cytoband Information From OneArray database. Cytoband disease related data for different segments of chromosomes. The data was integrated with other functional related data to process chunk annotation. (XLSX 29 kb)

Additional file 3: **Table S3.** List of Copy Number Variation in Clinvar Database. A collection of the Copy Number Variations in the clinvar database use for chunk annotation. (XLSX 779 kb)

Additional file 4: **Table S4**. List of Copy Number Variation in Disease Database (CNVD). (XLSX 1074 kb)

Additional file 5: **Table S5.** IGS Phenotype Information From the NCBI Map Viewer. Phenotype data integrated in IGS for each sample deposited herein. The data is extracted by use of the phenotype ID (key). (XLSX 350 kb)

Additional file 6: **Figure S1.** Pipeline Summary for Parametric Analysis of a single IGS Chunk. An experiment conducted to evaluate I/O dependency of two systems(Local disk and Maha) environment. Nine out of ten processes in the pipeline were used and the system characteristics results for each process was recorded. (DOCX 11651 kb)

Additional file 7: **Table S6.** List of Gene Boundaries in the Reference Genome (GRCh37). A file of chunk boundaries and the corresponding chromosome and gene of the region. (XLSX 1893 kb)

Additional file 8: **Table S7.** IGscan QC analysis for the first 1000 positions of the BRCA1 gene. An exemple of statistical analysis to show the advantage of using IGS to analysis a given genome region, BRCA 1 gene in this case. (XLSX 107 kb)

## Abbreviations

API: Application-programming interface
BAM: Binary alignment map
ETRI: Electronics and Telecommunications Research Institute
GWAS: Genome-wide association studies
HDD: Hard disk driver storage
HLA: Human leukocyte antigen
HP: Hewlett Packard
HPC: High-Performance Computing
NFS: Network file system
NGS: Next-generation sequencing
SGI: Silicon Graphics International
SNP: Single-nucleotide polymorphism
SSD: Solid State Drive Memory
WGS: Whole-genome sequencing
