# Supplementary material for: Integrated genome sizing (IGS) approach for the parallelization of whole genome analysis
Source: BMC Bioinformatics. 2018 Dec 3;19:462. doi: 10.1186/s12859-018-2499-1 (PMC6276166; doi:10.1186/s12859-018-2499-1)
Supplement: Supplementary file 6 — Figure S1. Pipeline Summary for Parametric Analysis of a single IGS Chunk. An experiment conducted to evaluate I/O dependency of two systems(Local disk and Maha) environment. Nine out of ten processes in the pipeline were used and the system characteristics results for each process was recorded. (DOCX 11651 kb) [file 12859_2018_2499_MOESM6_ESM.docx]

**Pipeline Summary for Parametric Analysis of a single IGS Chunk**

Nine out of ten pipeline processes (excluding step 1.Split) were used to evaluate the I/O characteristics in the Local disk & Maha environments. In the experiment, we estimated the average optimal I/O load for each system. Three different system parametric settings were implemented; para 1: 4cores/20GB memory, para 2: 8cores/30GB memory and para 3: 8cores/64GB memory. The para 3 setting was default in both systems. However, para 1 and para 2 were unique settings in the Local disk and Maha respectively. Four experimental cases were chosen studied:

**Local disk 🡪 Case 1:** 4cores/20GB and **Case 2:** 8cores/64GB (default)

**Maha** (distributed parallel computing) **🡪 Case 3:** 8cores/30GB and **Case 4:** 8cores/64GB (default)

A 1GB network was available across all processes. The amount of memory used and the recorded I/O characteristics at each step was different. For instance 10GB & 11GB of memories were used for 4cores/20GB and 8cores/64GB in the local disk environment. On the other hand, 6GB of memory were both used for the 8cores/30GB and 8cores/64GB in the maha environment. It was further confirmed that an average of 12GB was utilized for the case 1 and case 3 respectively even though the amount of memory available was large. For optimum performance, the 4cores/12GB parameter condition was recorded and further memory increment yielded no significant gain. Below is a concise characteristic result for the nine processes.

**The I/O Characteristics of each Pipeline Process (1. Split ~ 10. GATK-Haplotype Caller) in the Local disk and Maha Environments.**

**Split (Step 1)**

No system graphical output pertaining to this step was available. Unlike the other nine pipeline processes, the split merely involves splitting whole genomes to chunk. Thus I/O characteristics was not feasible as oppose to the rest of the steps.

**Sickle (step 2)**

**LOCAL DISK**

**MAHA**

**Case 1: 4cores/20GB memory**

**Case 2: 8Cores/64GB memory**

**Case 3: 8Cores/30G memory**

**Case 4: 8Cores/ 64GB memory**


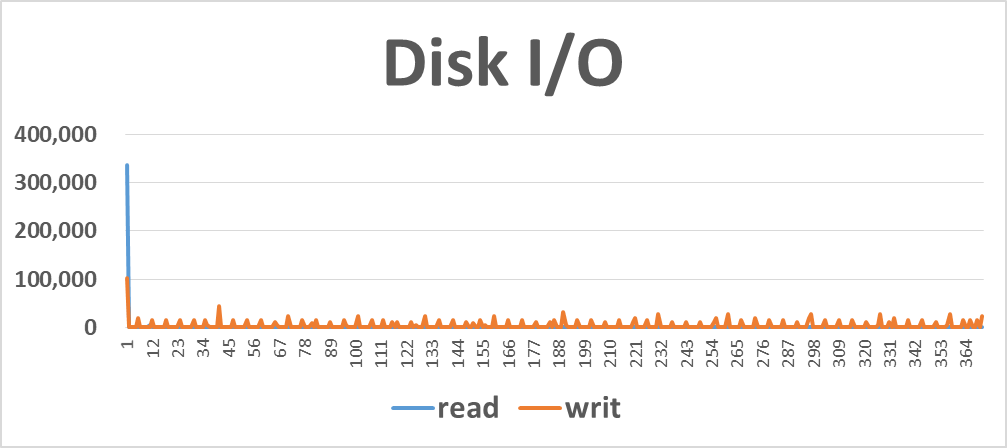

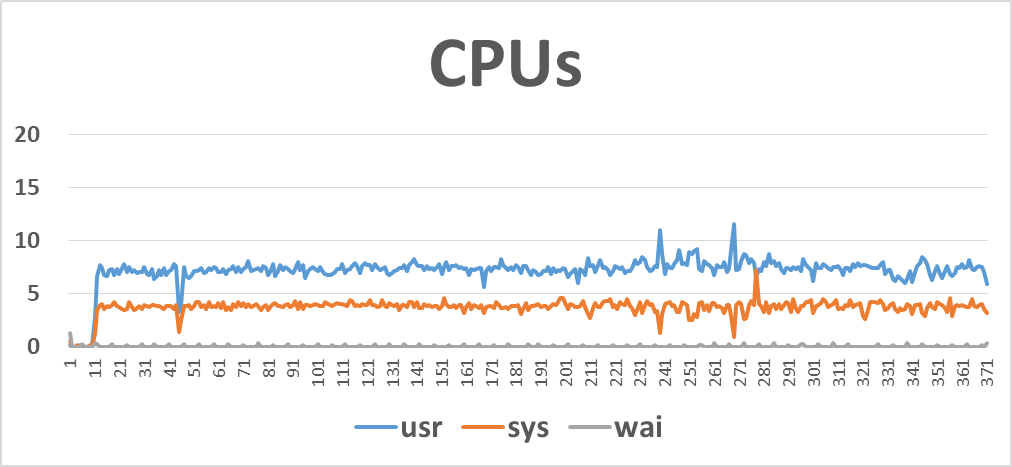

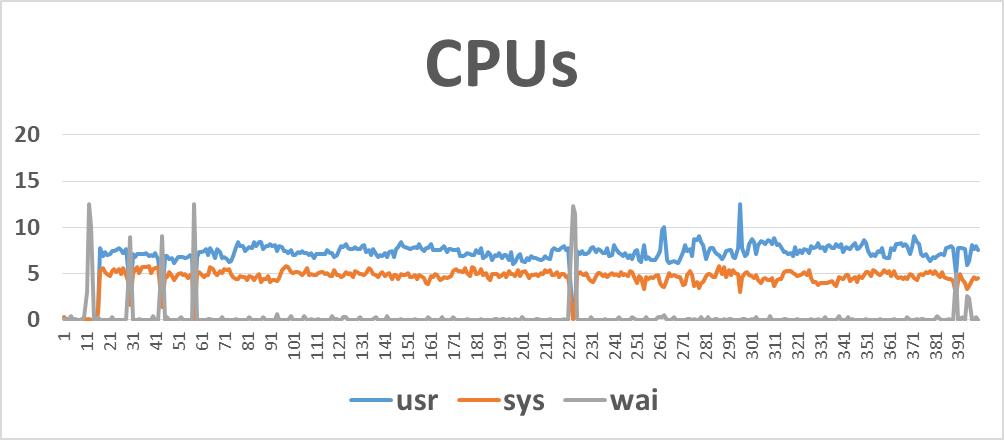

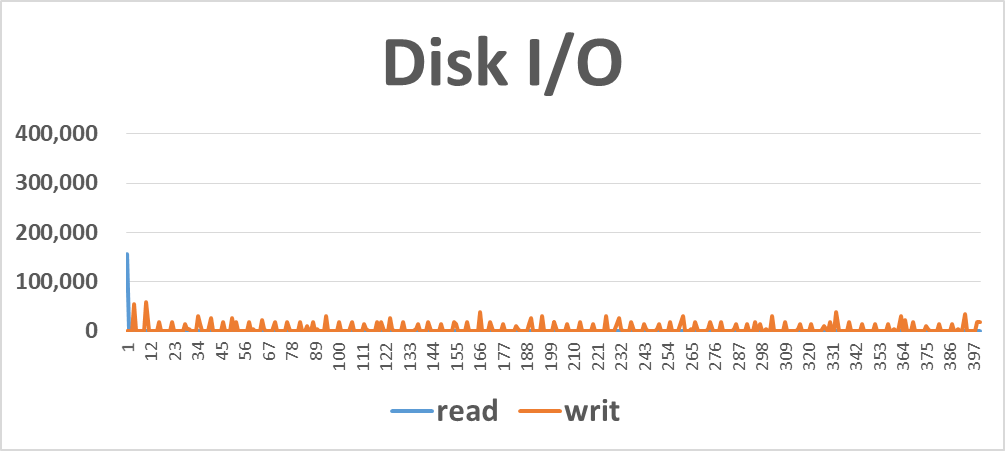

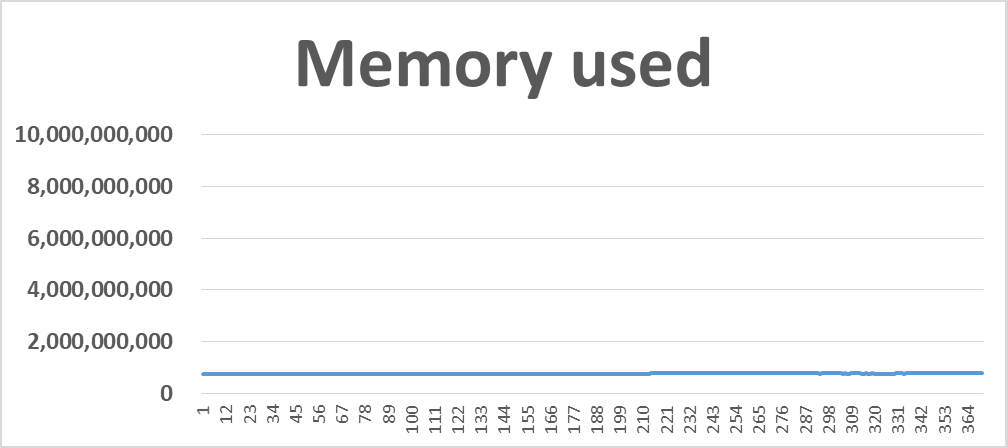

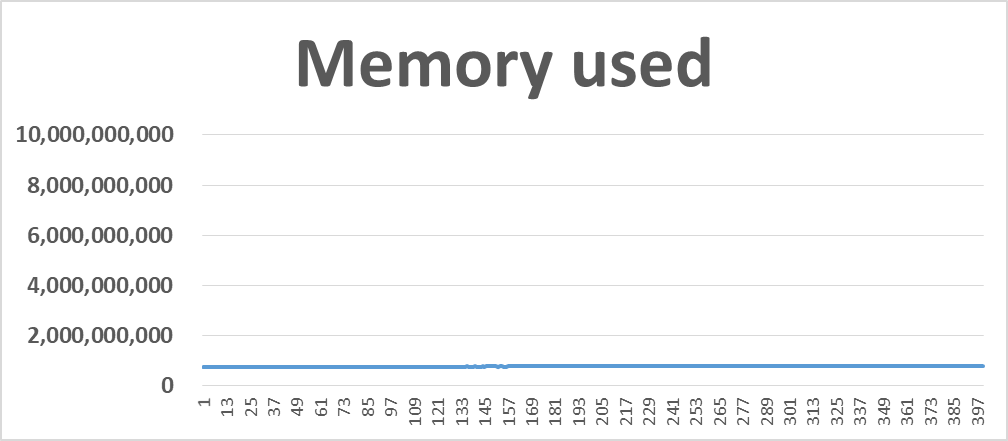

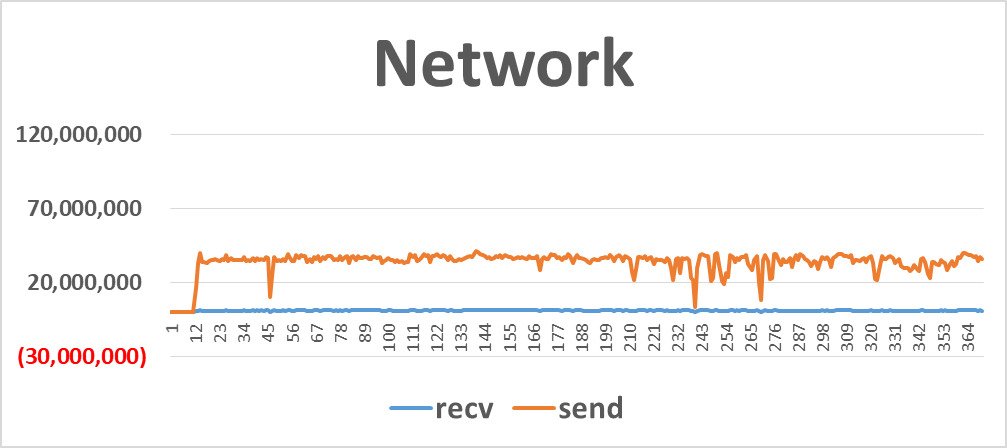

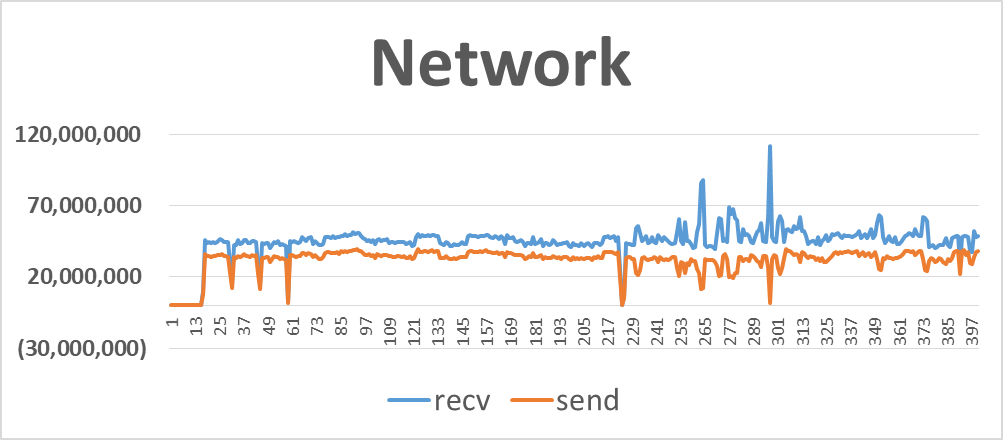


**30MB write**

**1GB**

**50MB read/ 40MB write**

**100%**

**8core**

**1GB**


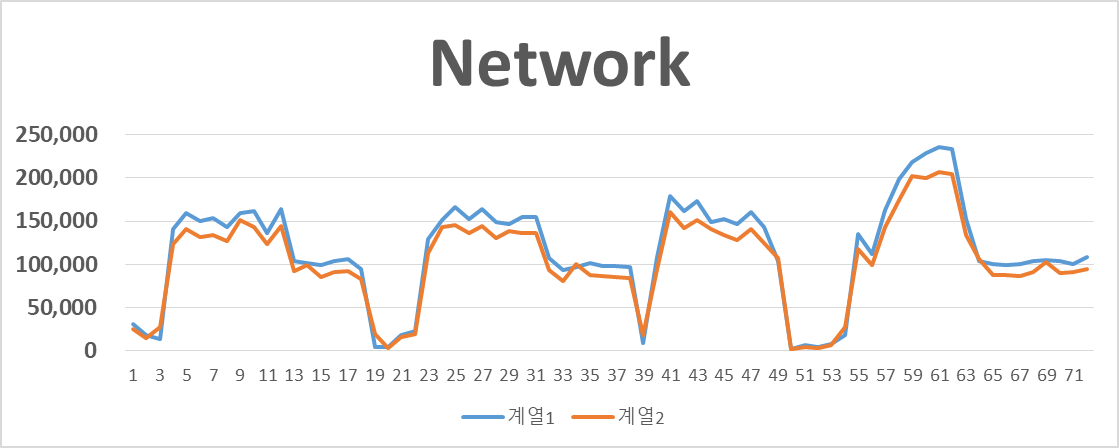

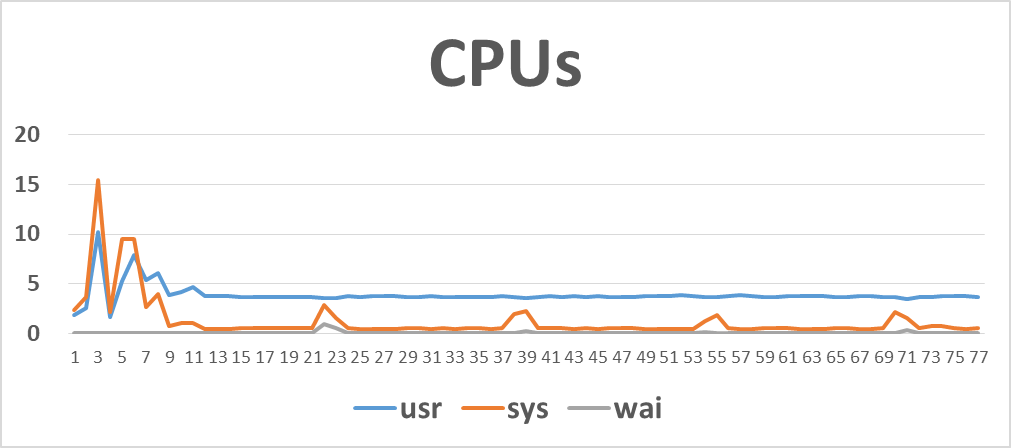

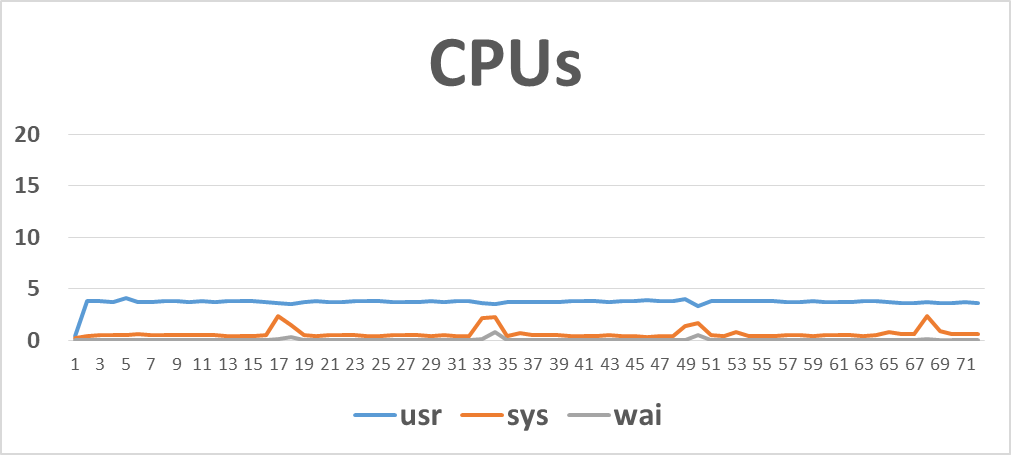

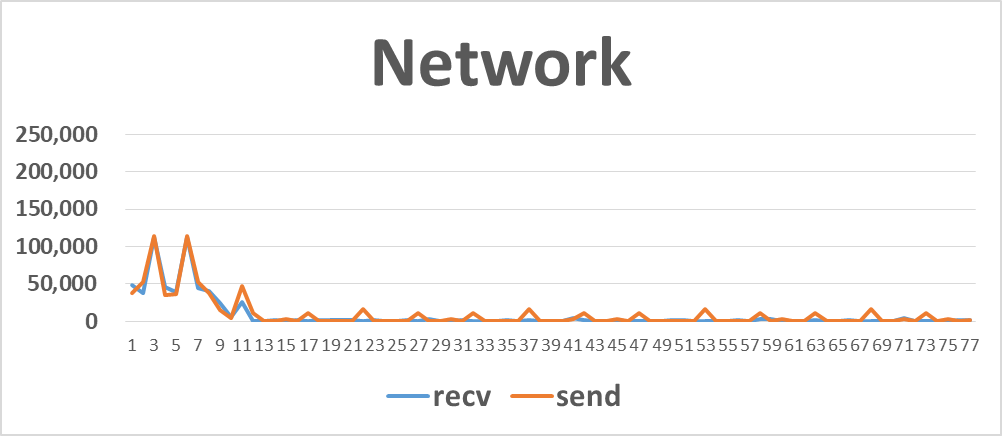

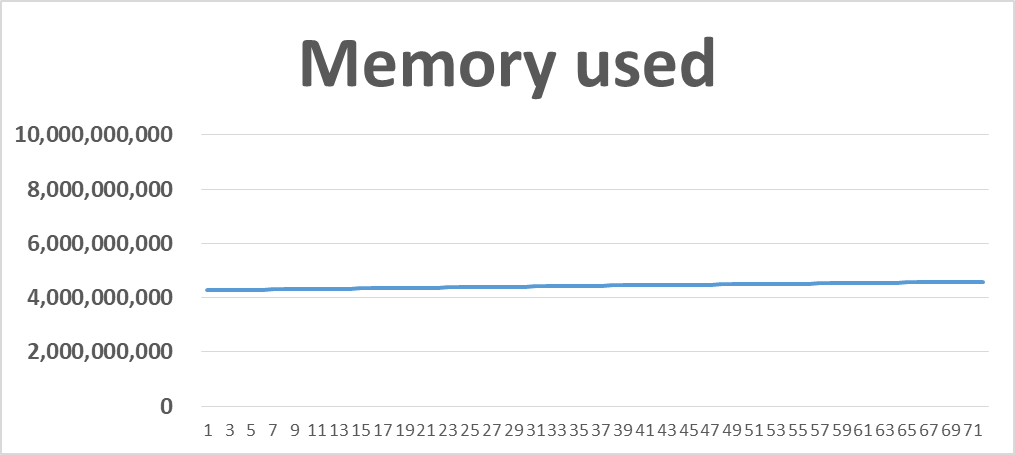

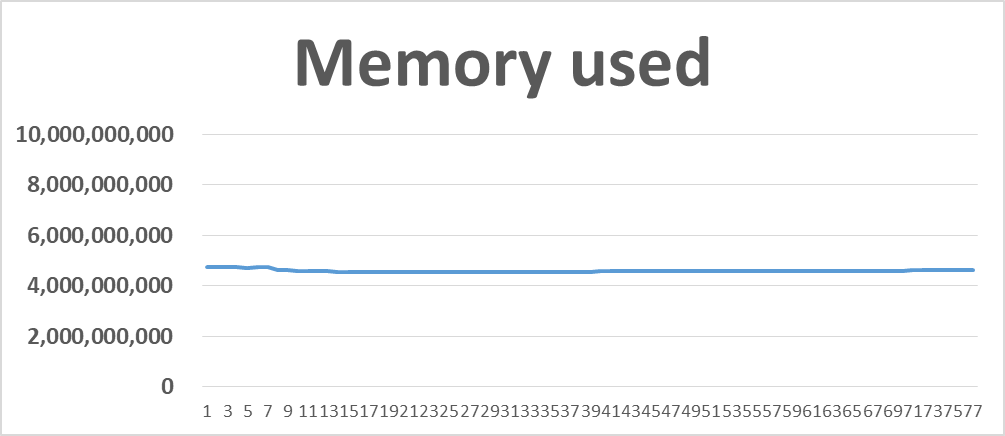

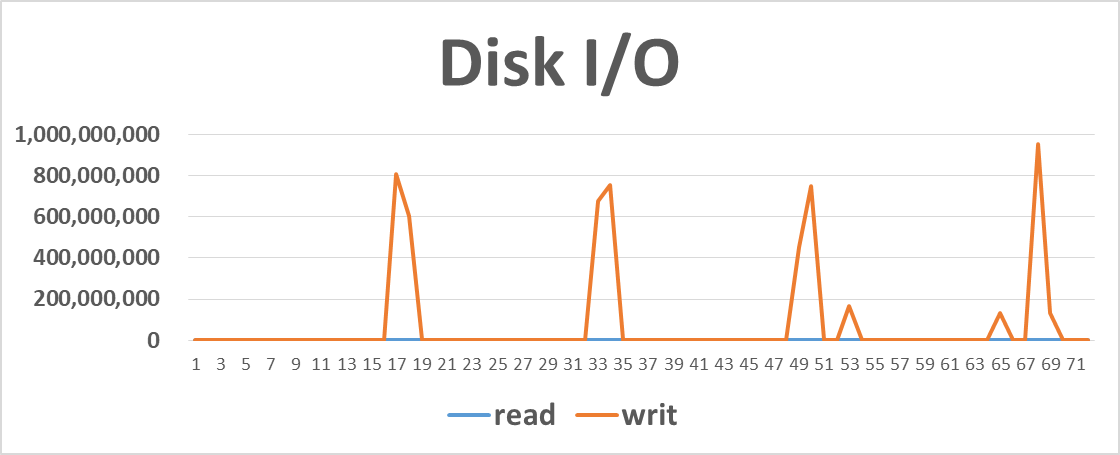

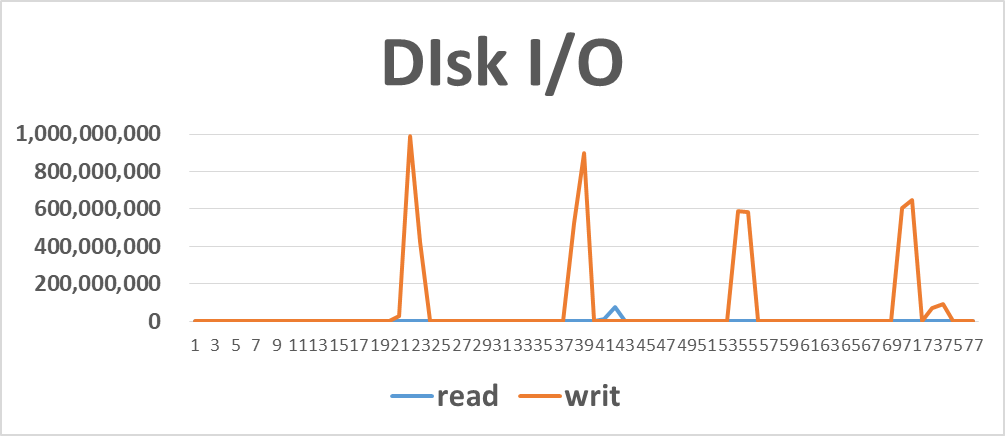


**4GB**

**100%**

**24core**

**10GB**

**1GB**

**4GB**

**BWA-MEM (Step 3)**

**LOCAL DISK**

**MAHA**

**Case4-8Core with 64GB memory**

**Case 4: 8Cores/ 64GB memory**

**Case 3: 8Cores/30G memory**

**Case 2: 8Cores/64GB memory**

**Case 1: 4cores/20GB memory**


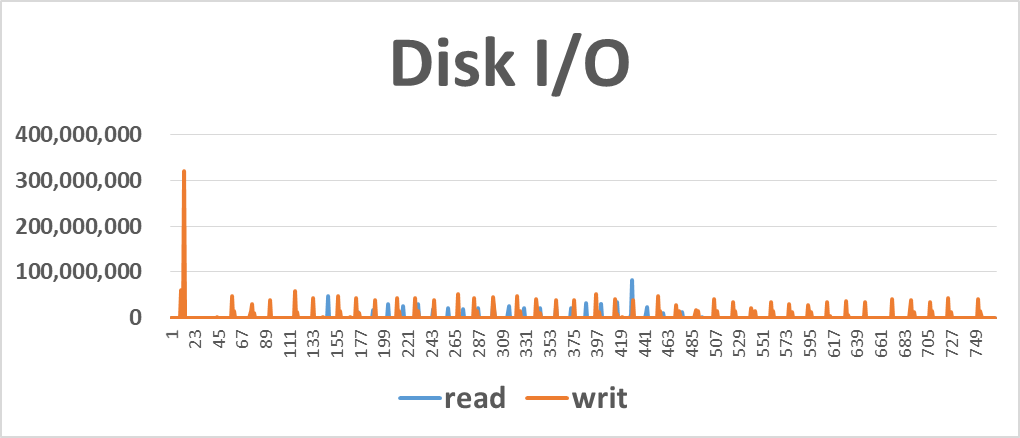

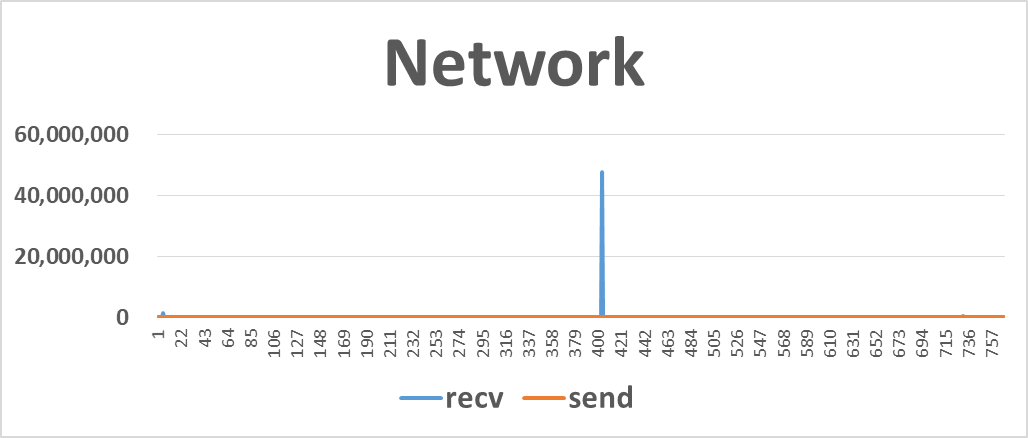

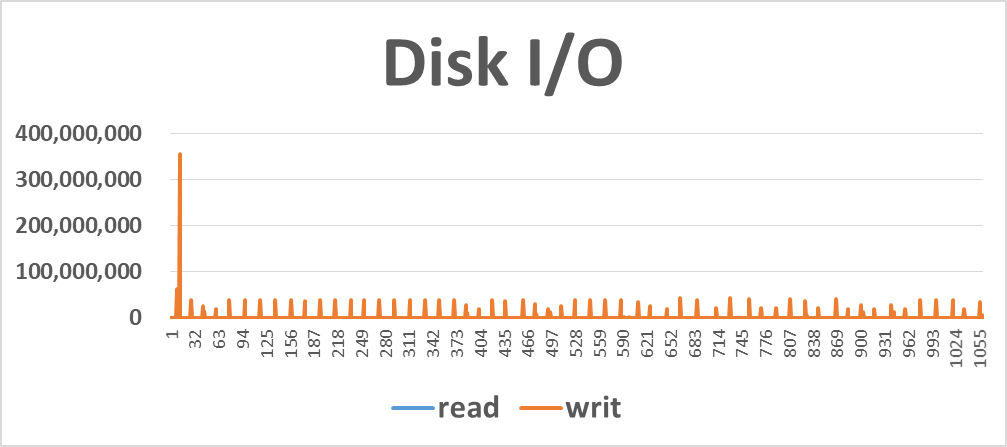

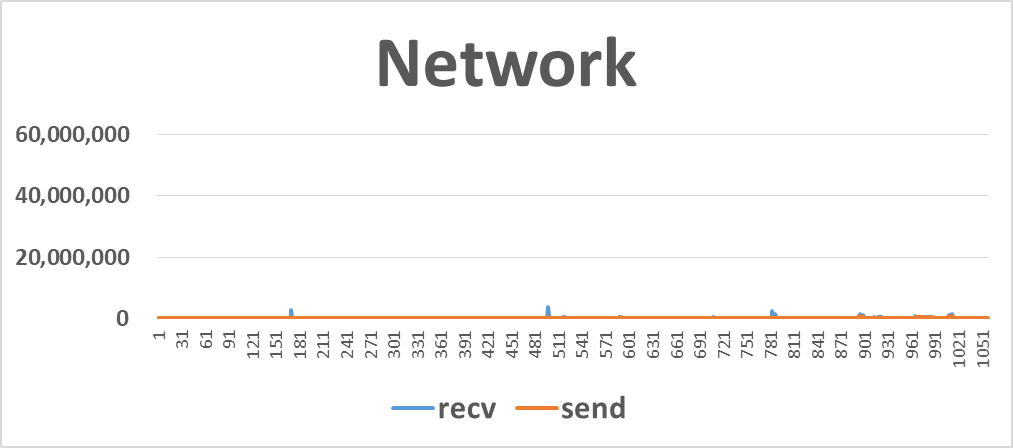

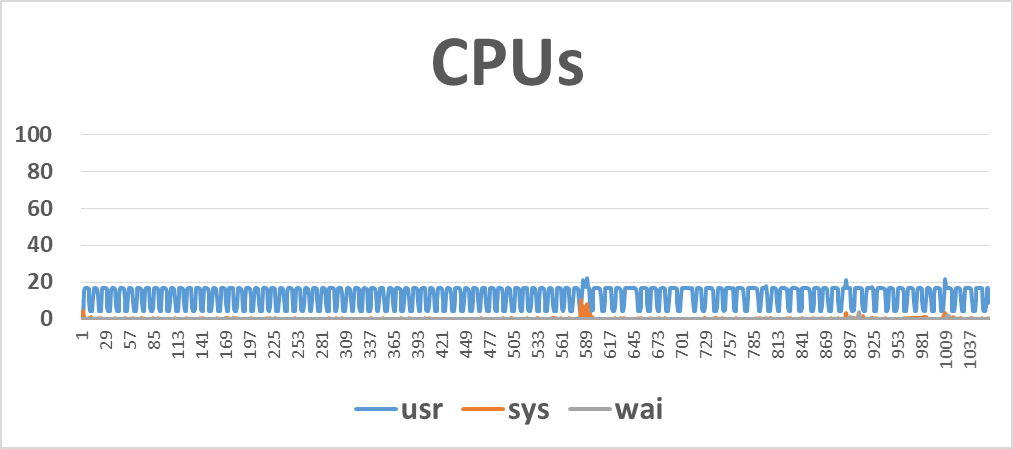

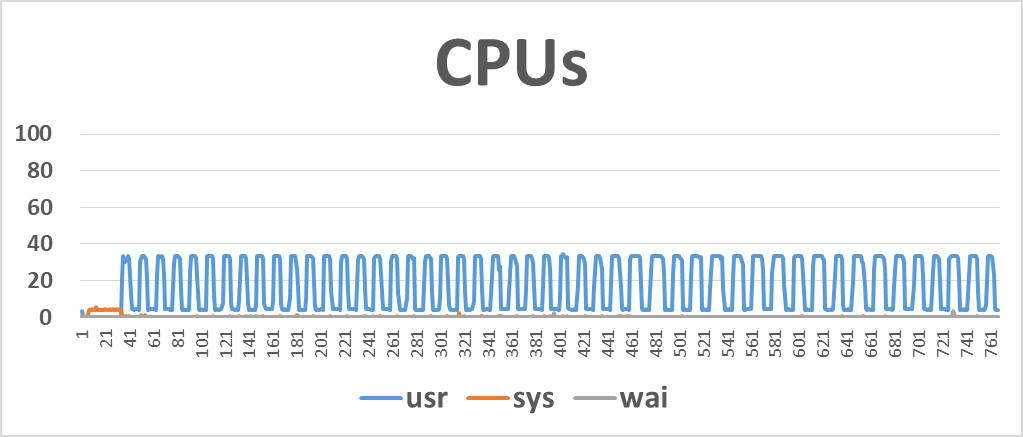

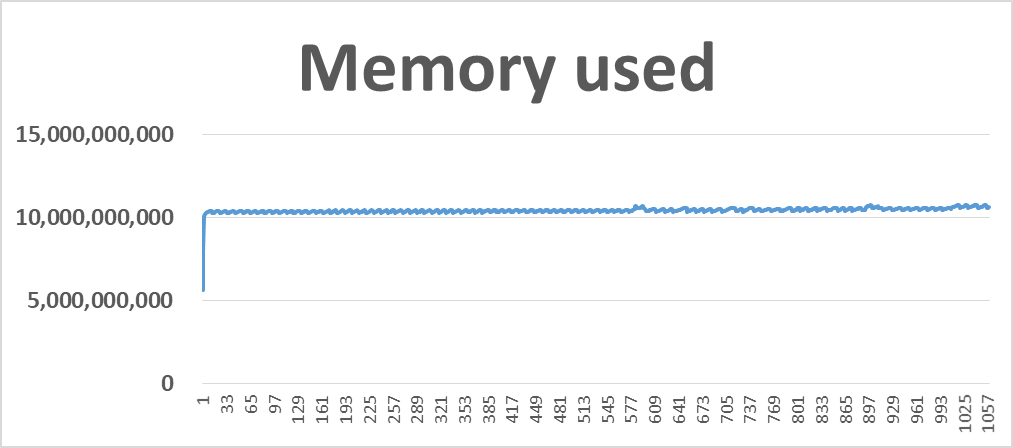

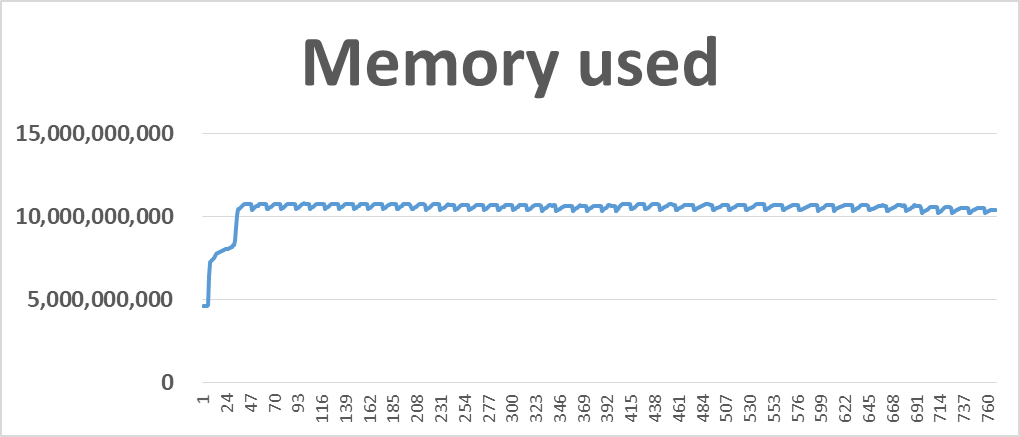


**11GB used**

**10GB used**

**4cores**

**8core**s

**40MB/sec write**

**40MB/sec write**


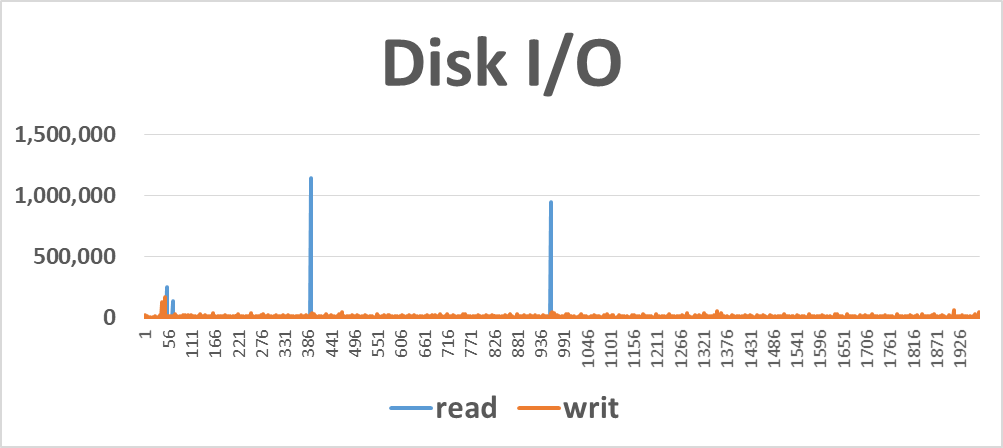

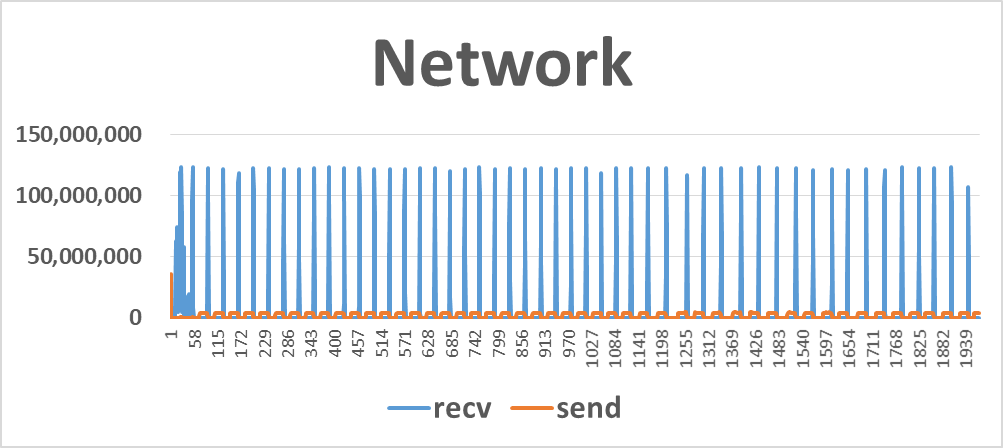

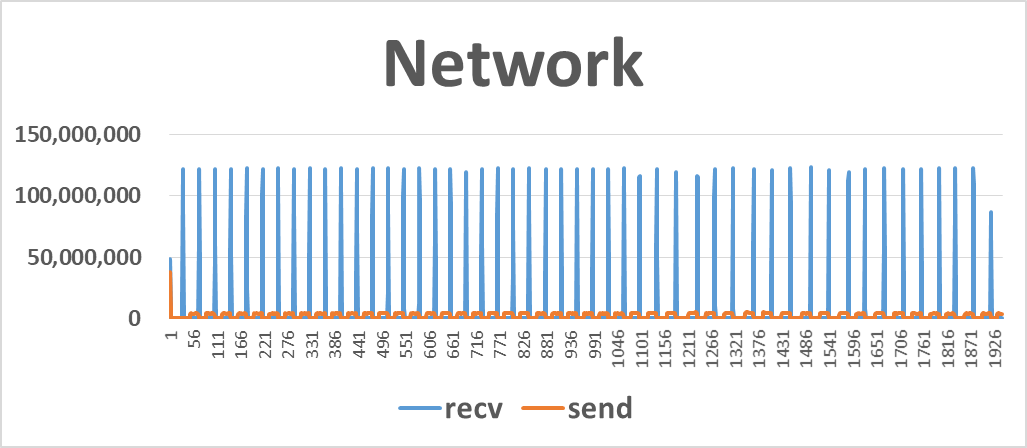

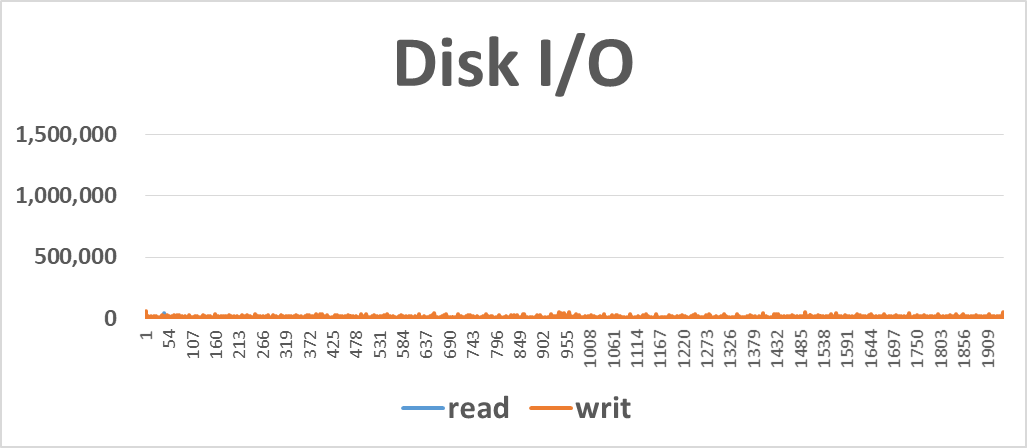

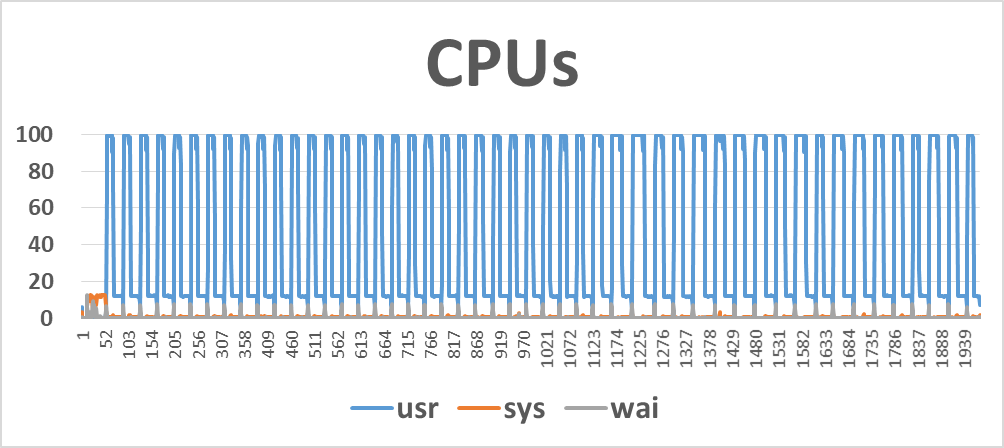

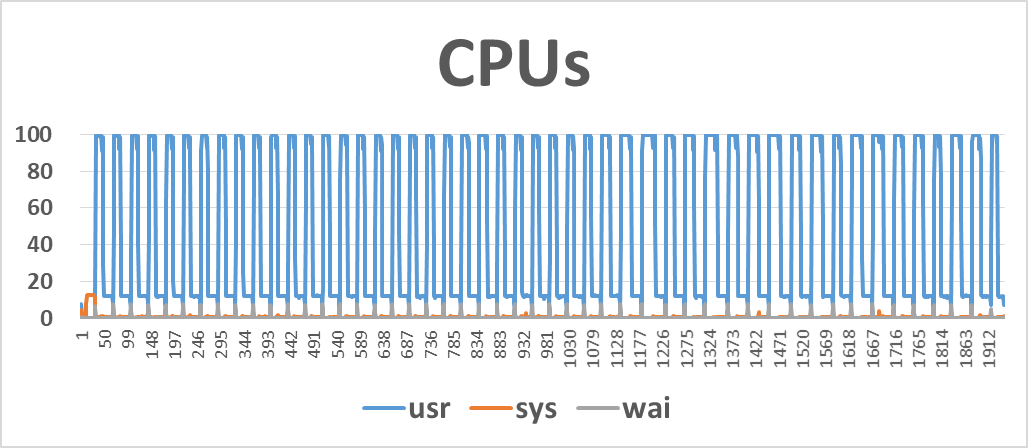

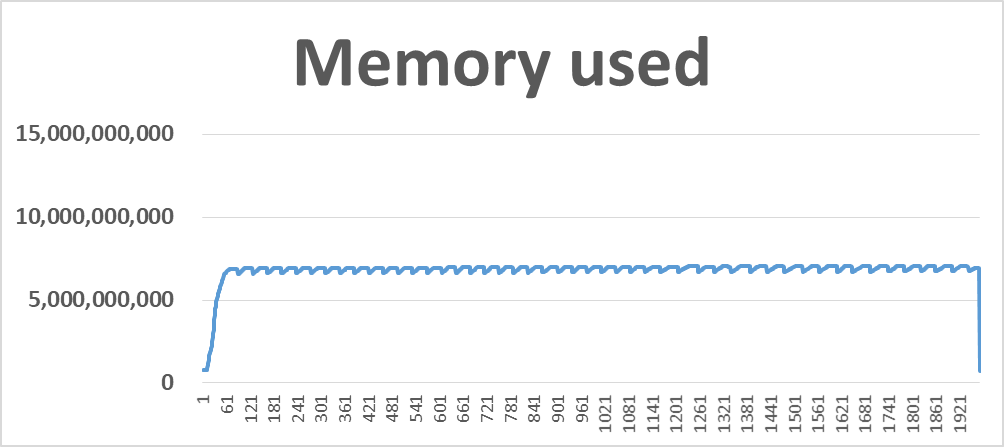

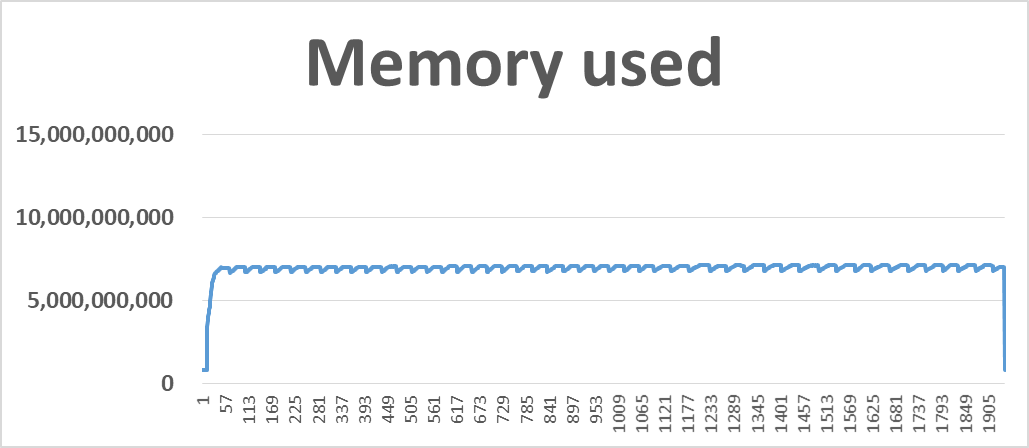


**6GB used**

**6GB used**

**8cores Max**

**8core Max**

**120MB/read Max**

**120MB/read Max**

**100%**

**8core**

**100%**

**8core**

**Case4-8Core with 64GB memory**

**Picard-Fix-Mate Information (Step 4)**

**LOCAL DISK**

**MAHA**

**1core used**

**Case 3: 8Cores/30G memory**

**Case 4: 8Cores/ 64GB memory**

**Case 2: 8Cores/64GB memory**

**Case 1: 4cores/20GB memory**


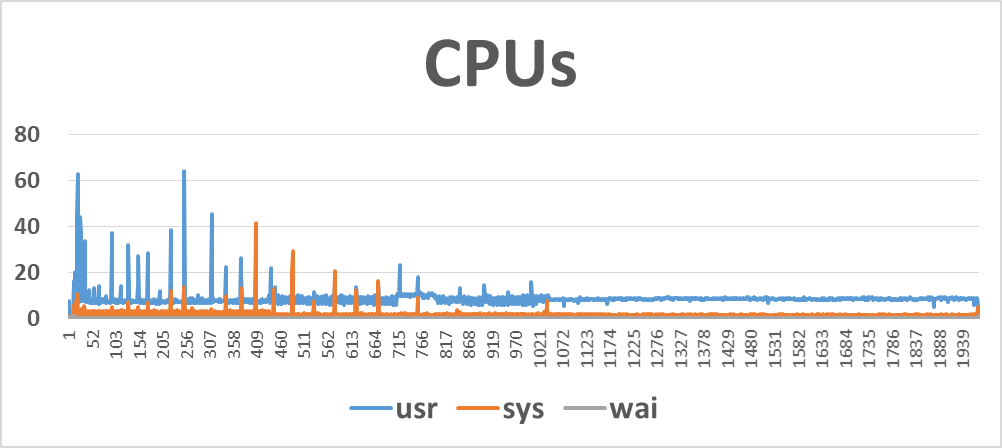

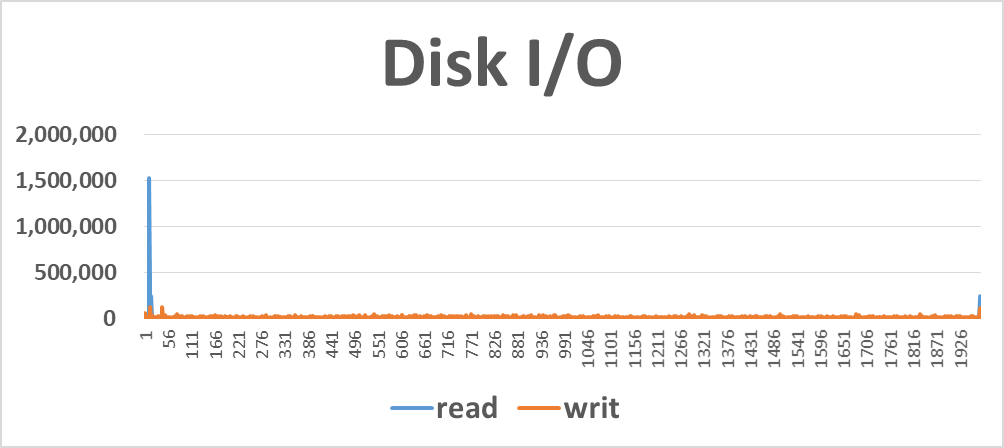

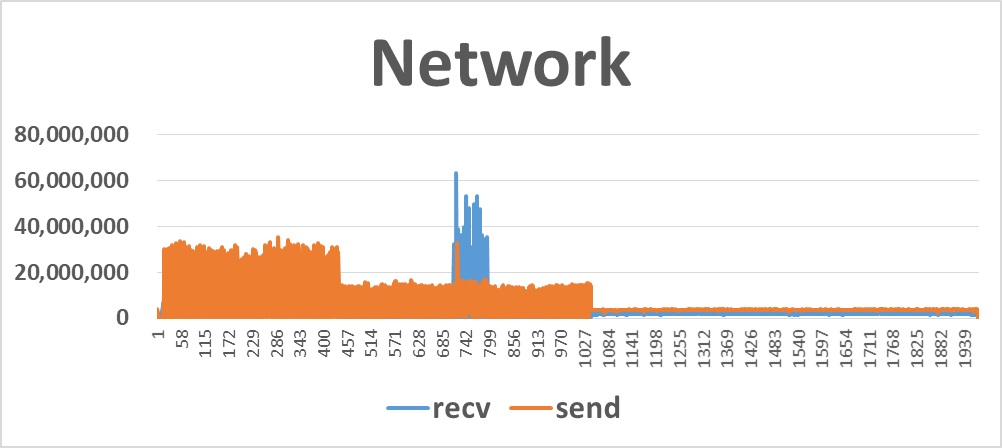

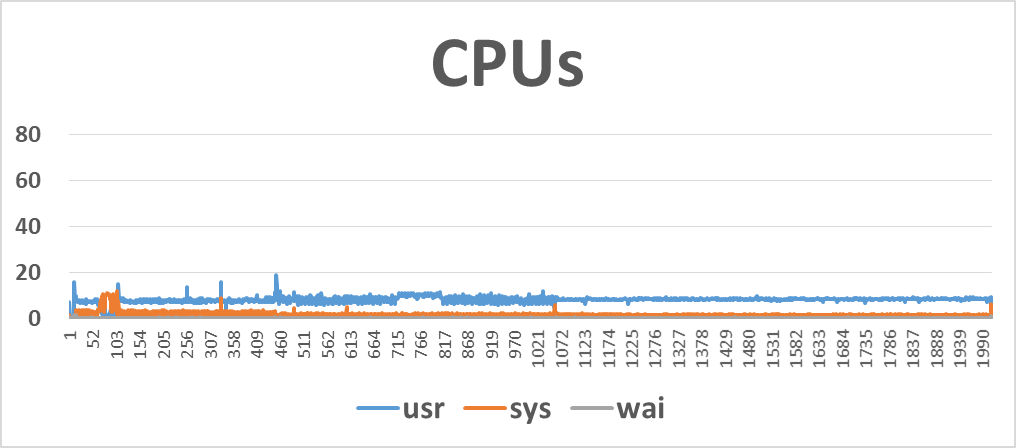

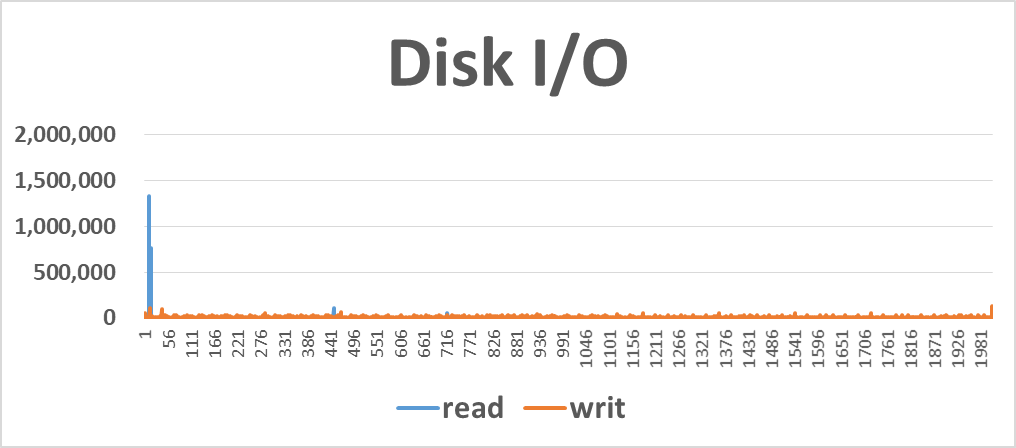

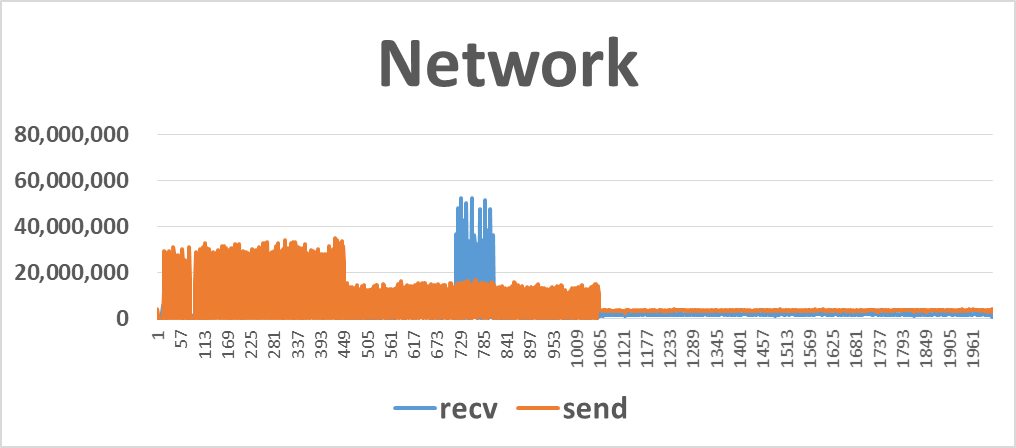

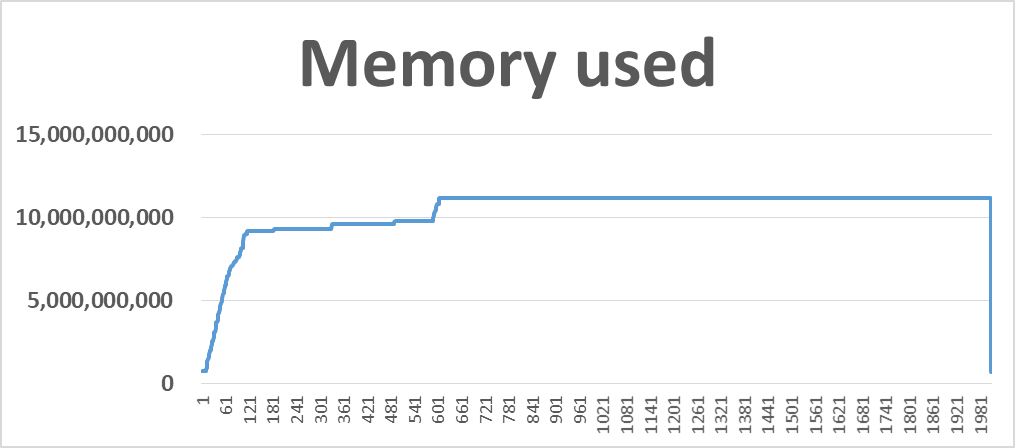

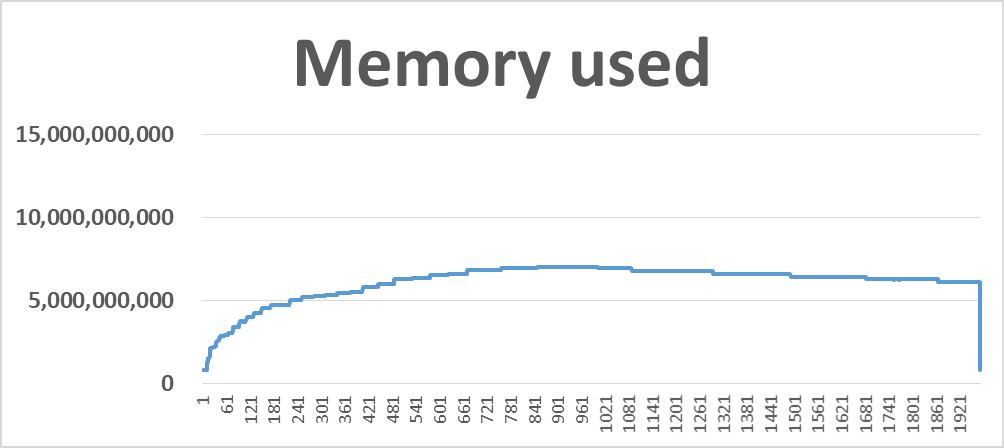


**35MB/sec write**

**11GB used**


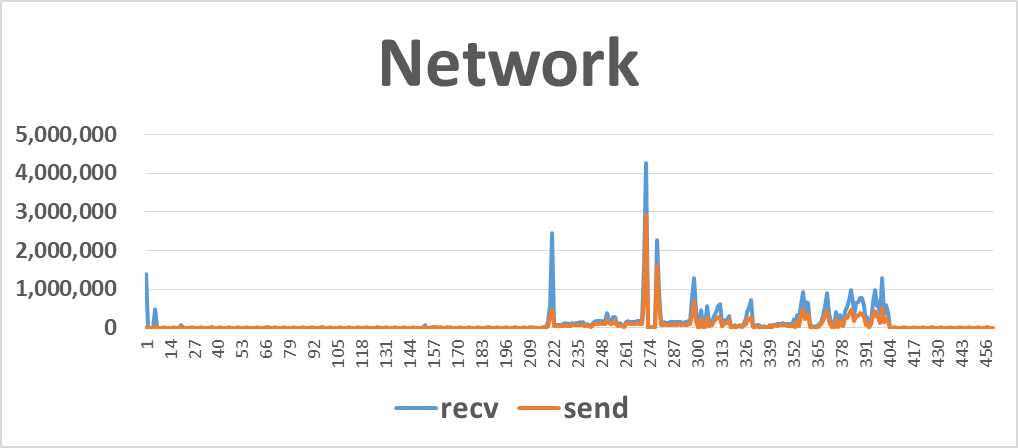

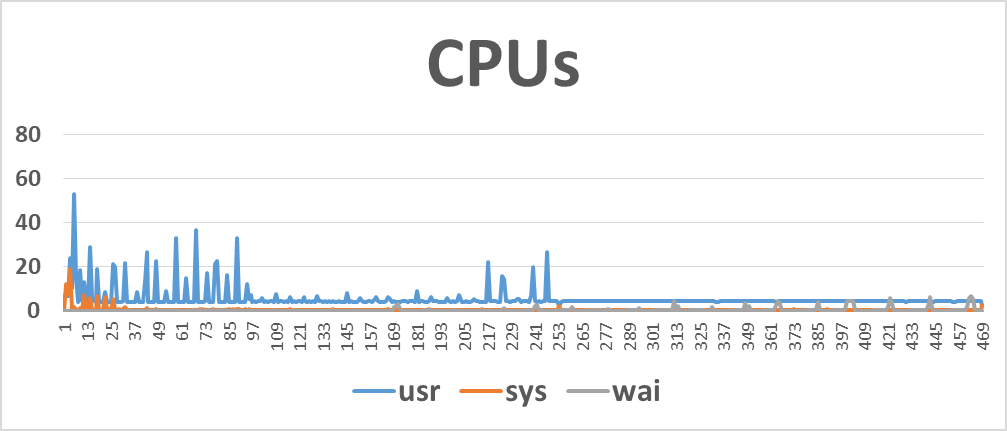

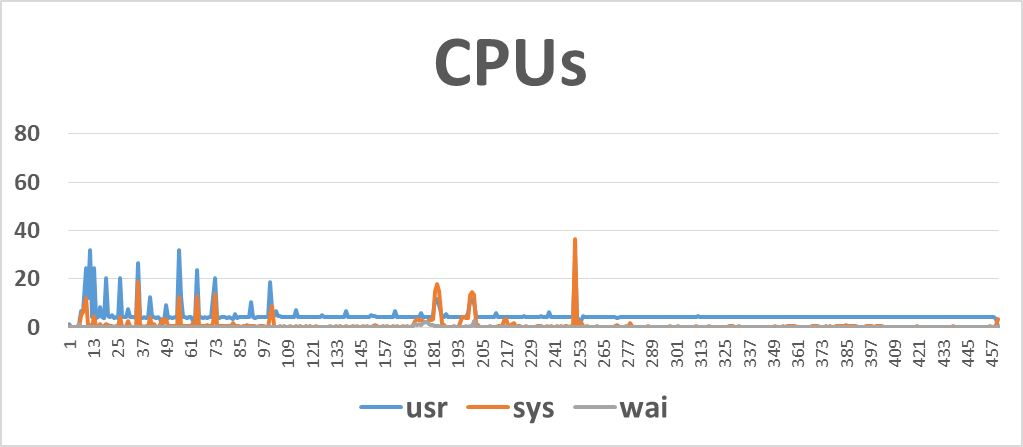

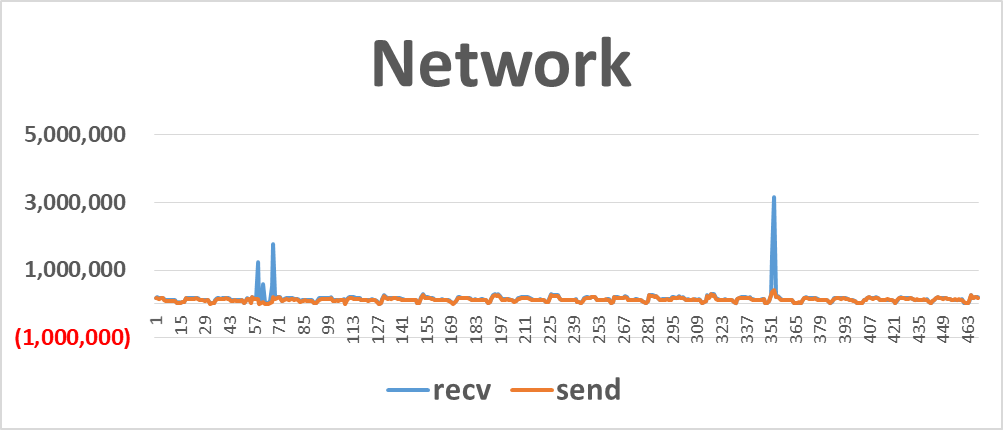

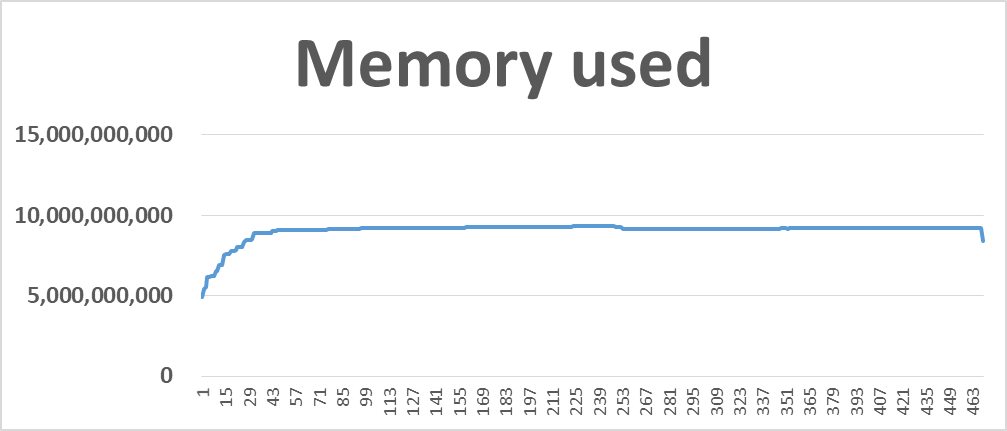

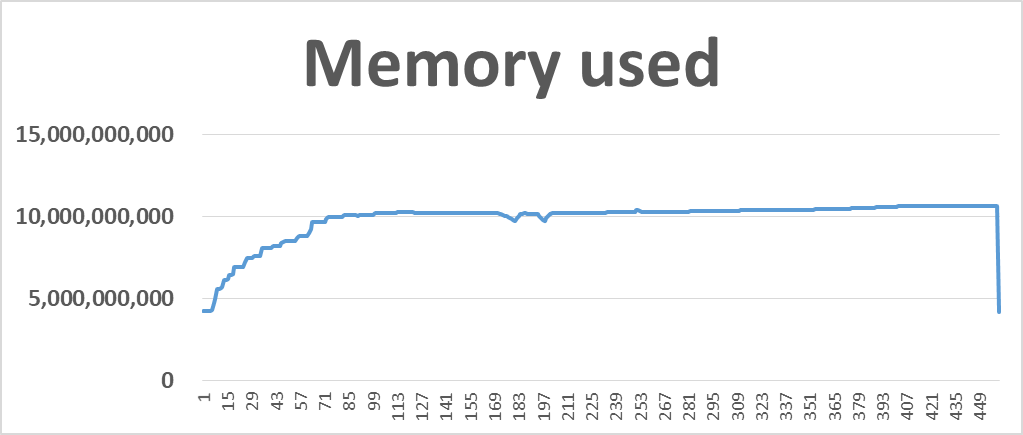

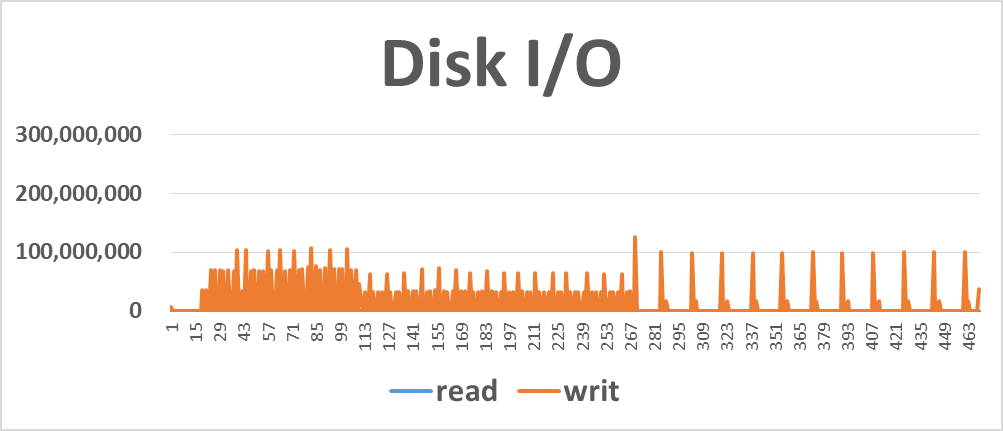

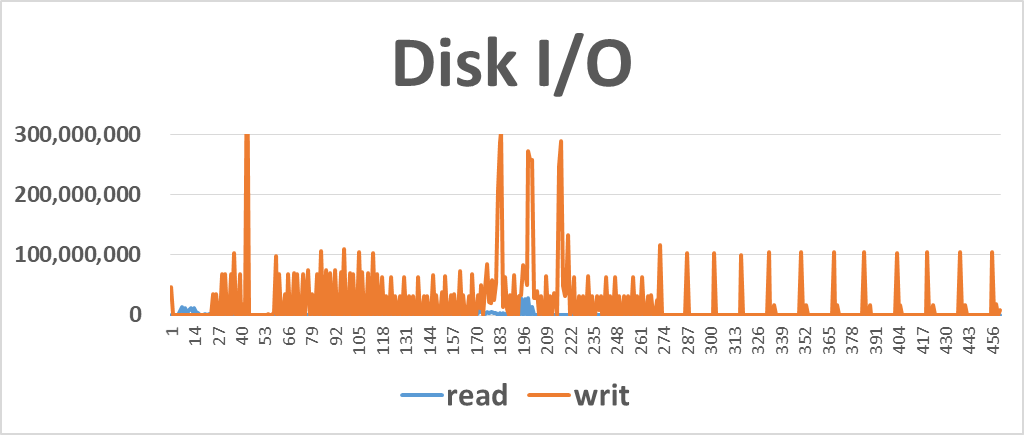


**9GB used**

**110MB/sec write**

**Picard-Mate Duplicates (Step 5)**

**Case 4: 8Cores/ 64GB memory**

**Case 3: 8Cores/30G memory**

**Case 2: 8Cores/64GB memory**

**Case 1: 4cores/20GB memory**

**LOCAL DISK**

**MAHA**


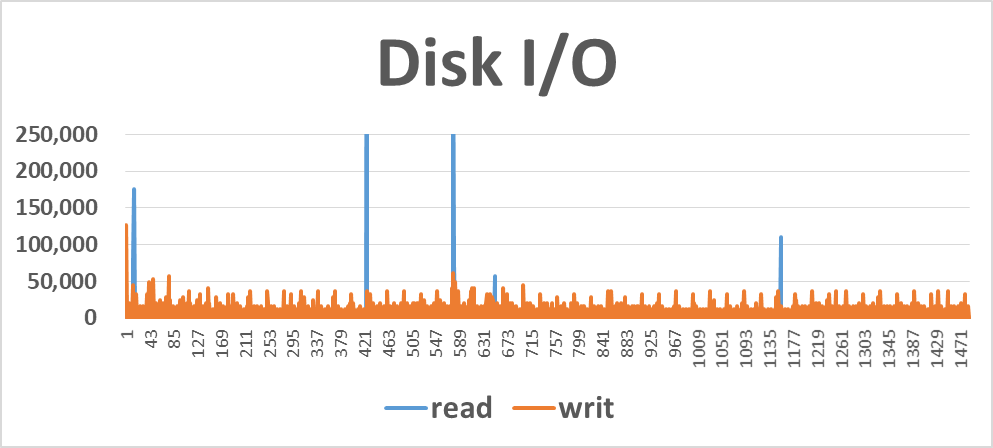

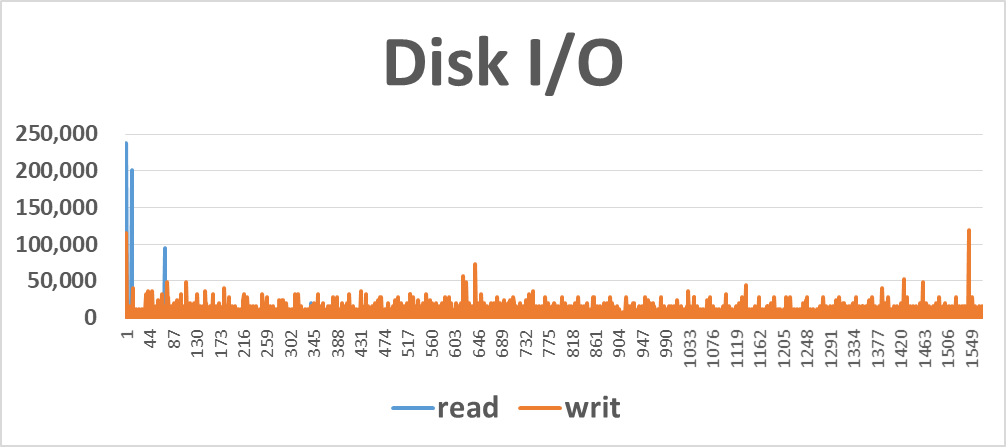

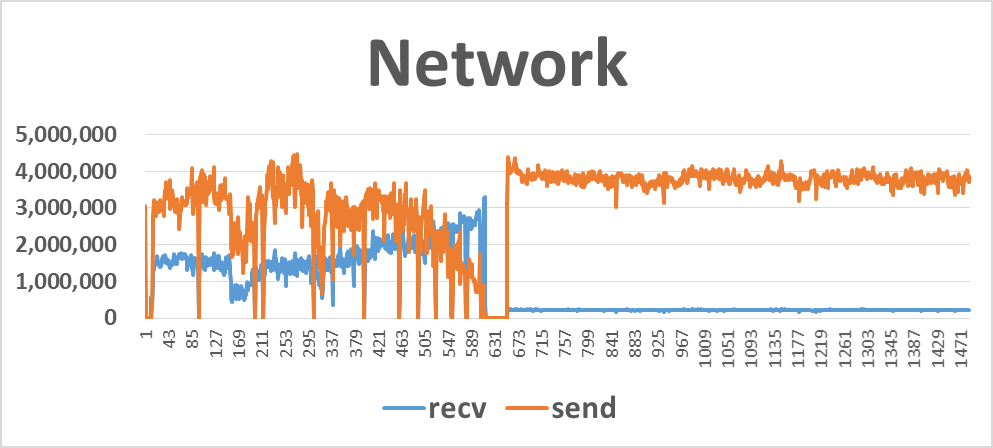

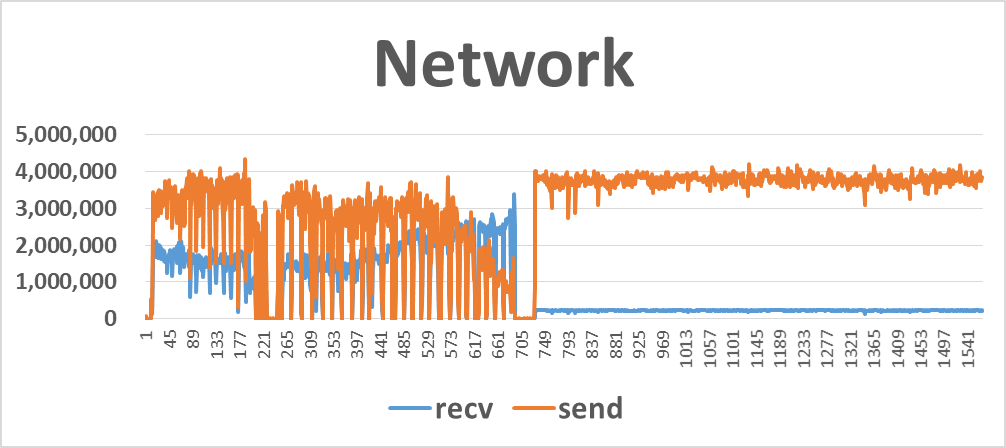

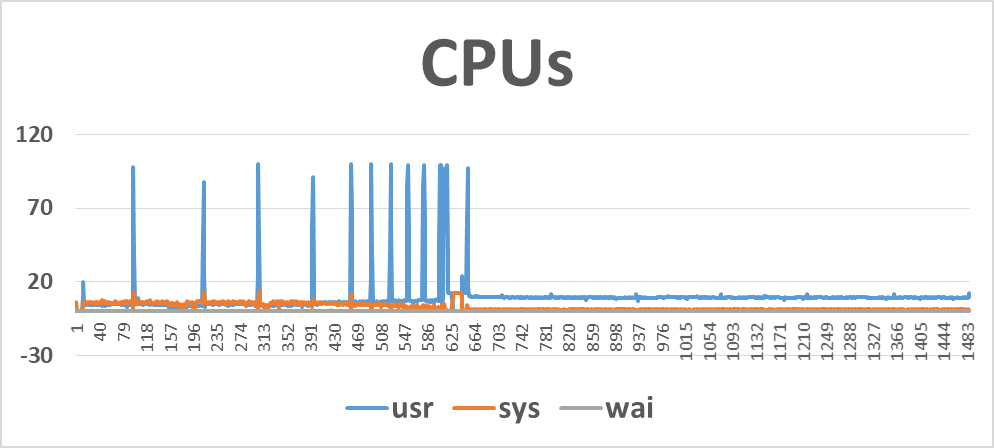

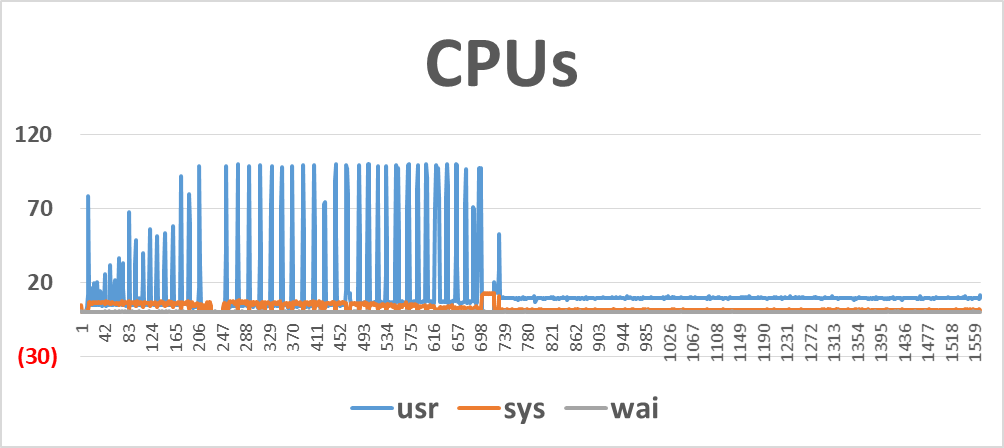

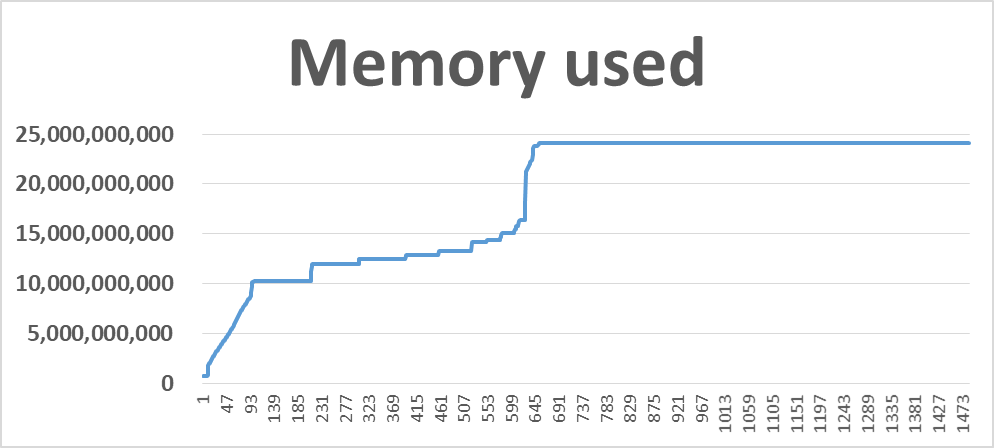

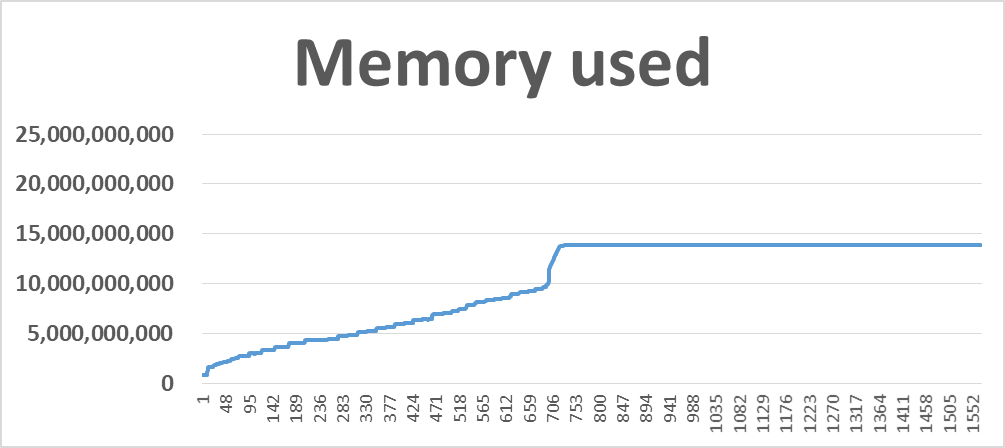


**4MB/sec write**

**24GB used**


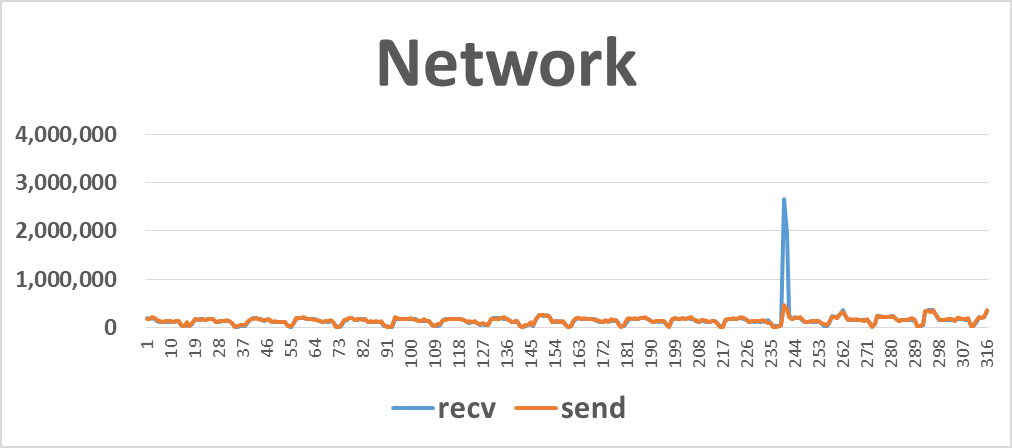

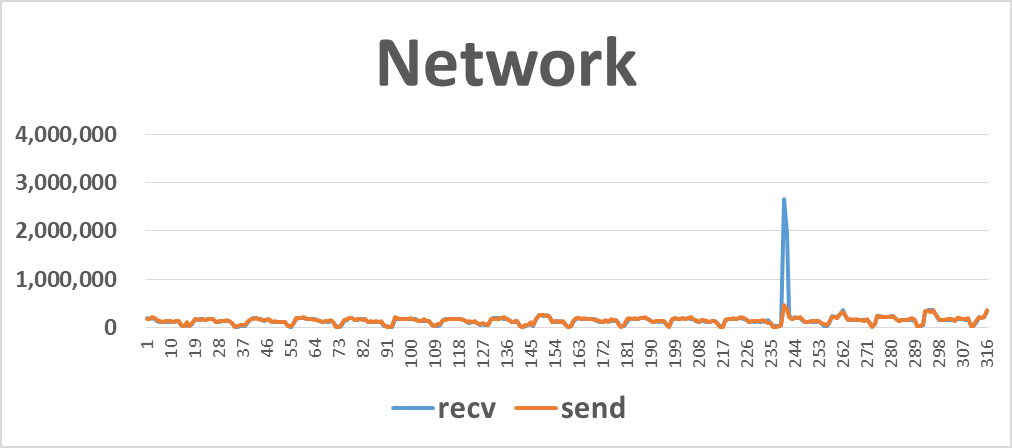

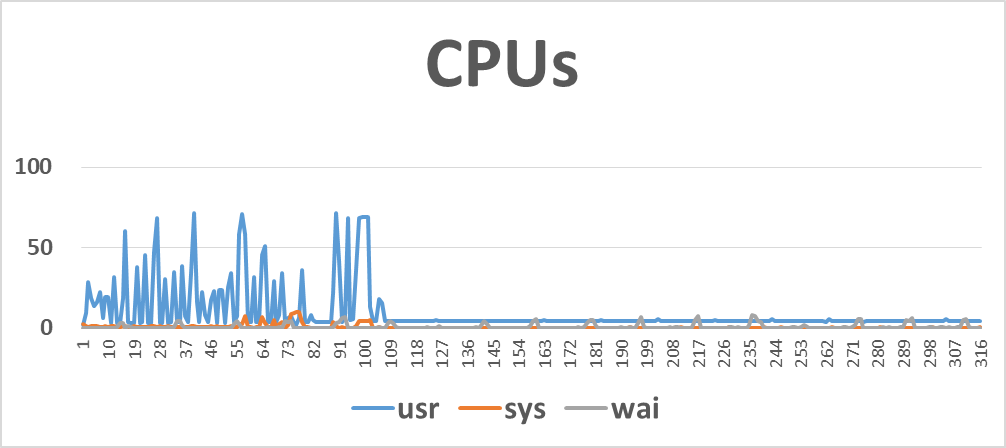

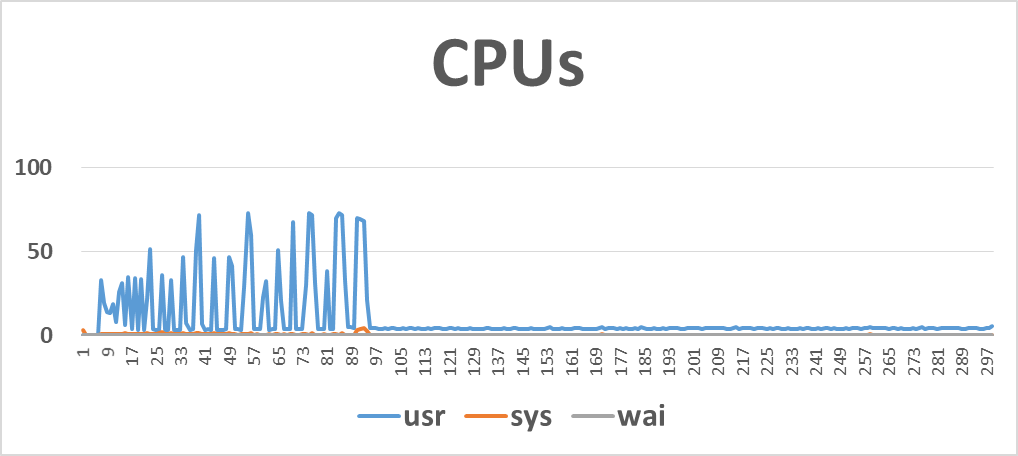

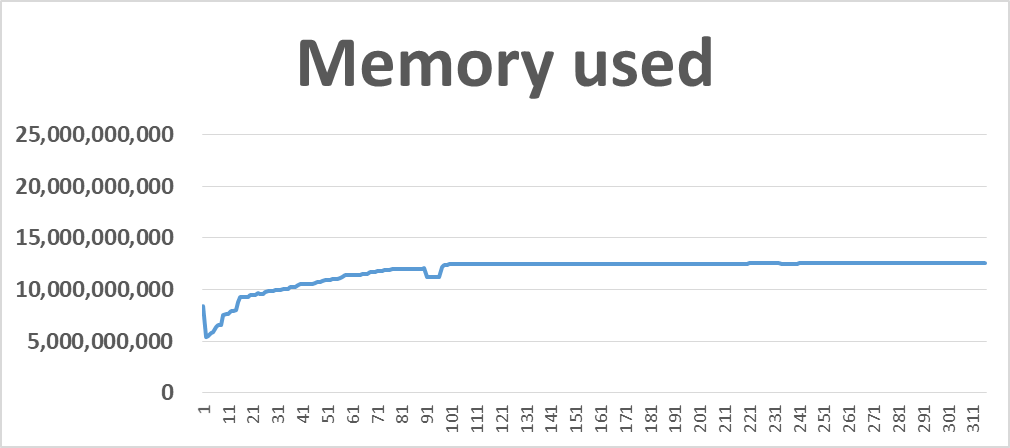

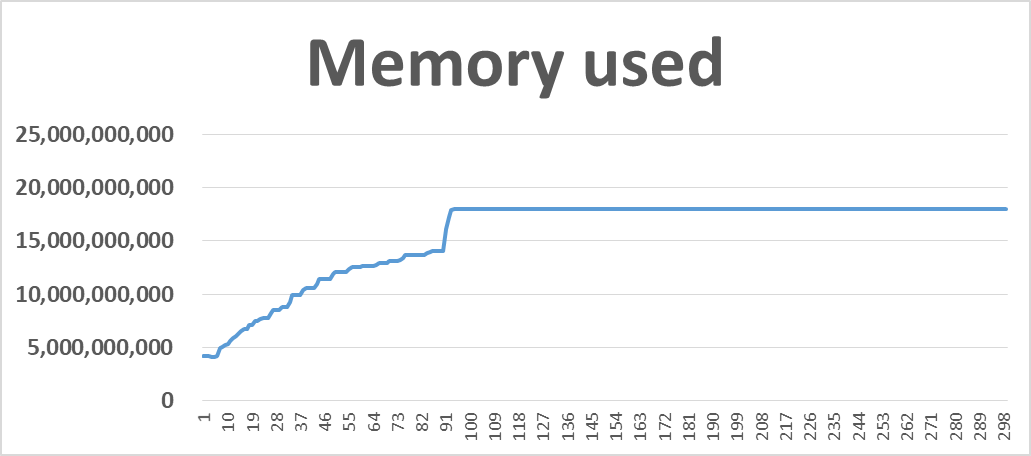

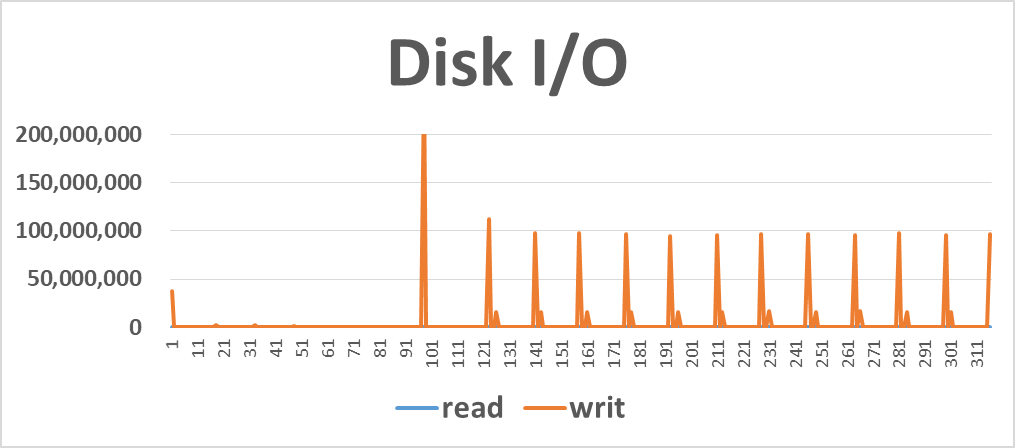

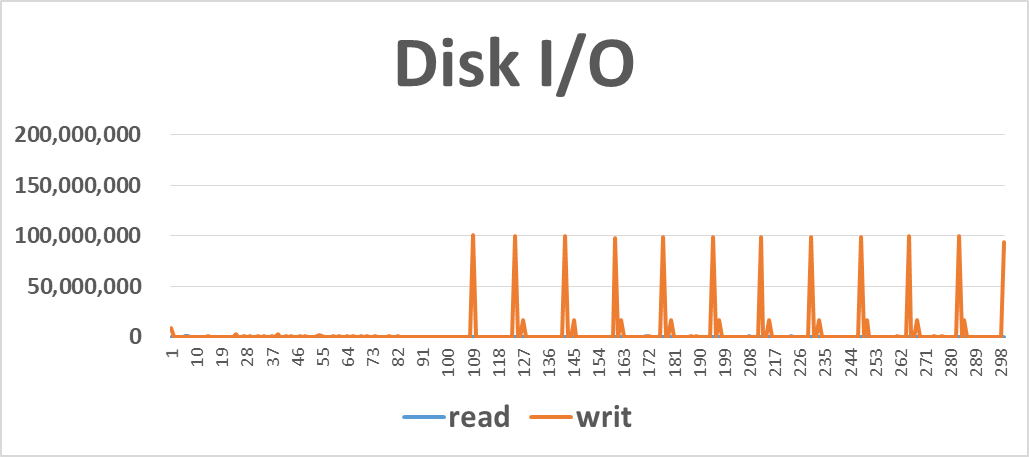


**19GB used**

**GATK-Realigner Target Creator (Step 6)**

**LOCAL DISK**

**MAHA**

**Case 1: 4cores/20GB memory**

**Case 2: 8Cores/64GB memory**

**Case 3: 8Cores/30G memory**

**Case 4: 8Cores/ 64GB memory**


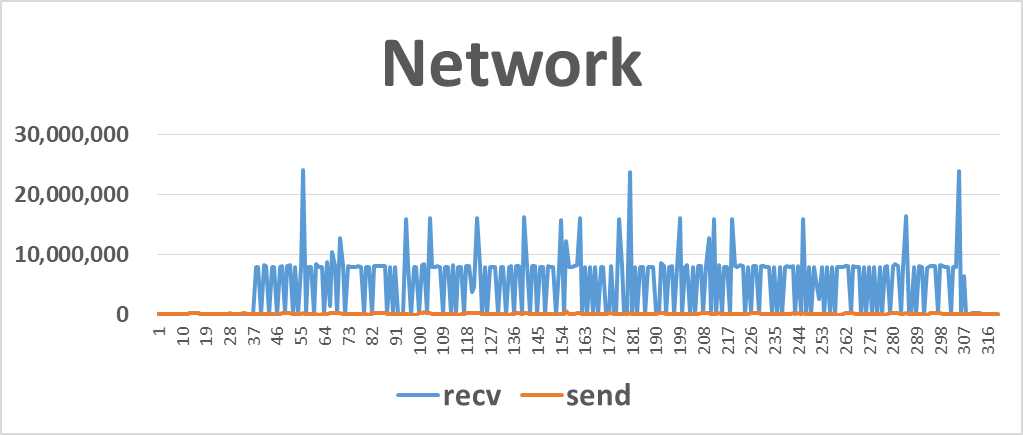

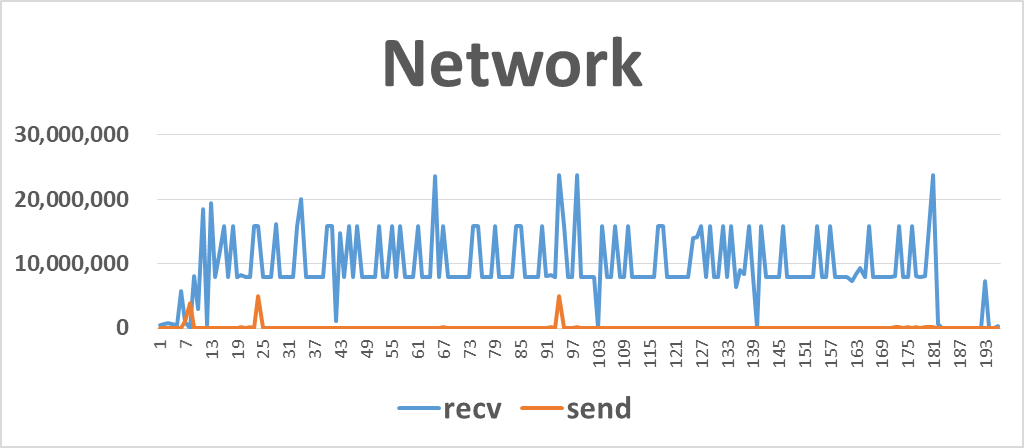

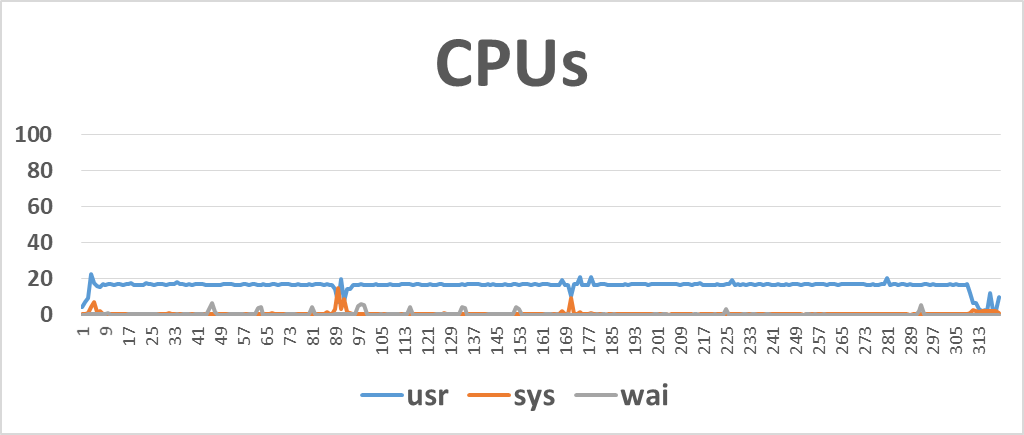

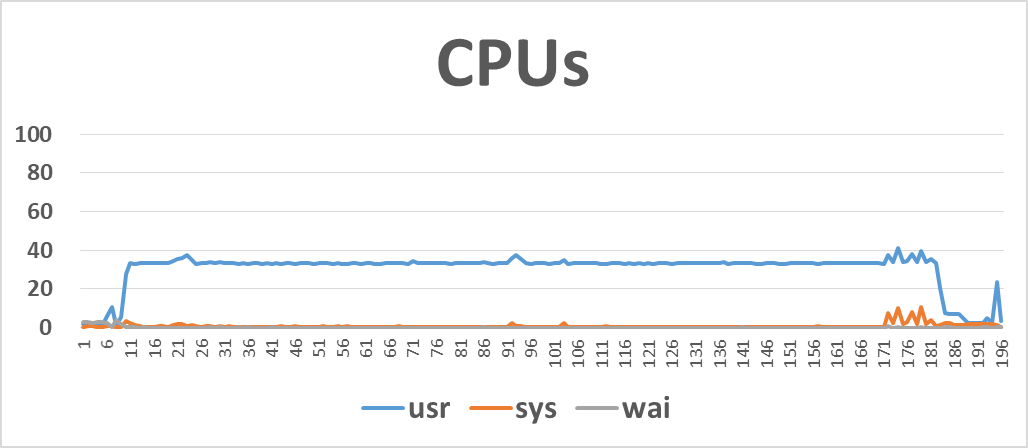

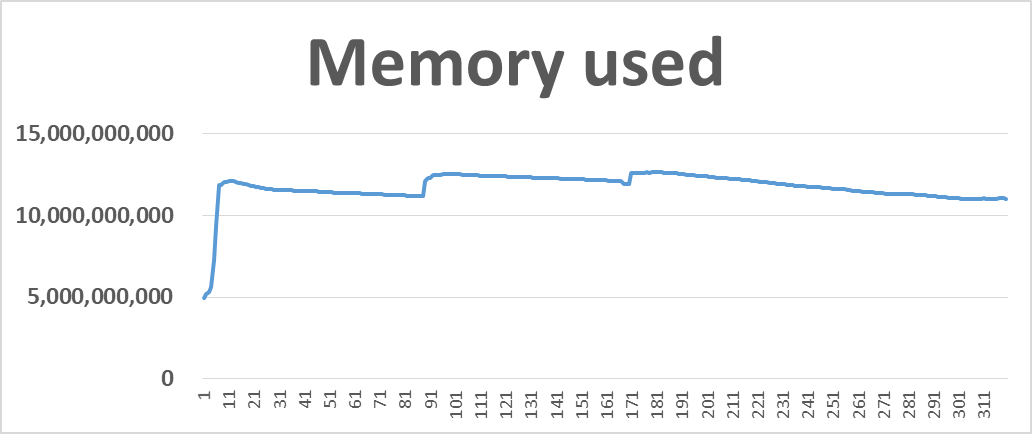

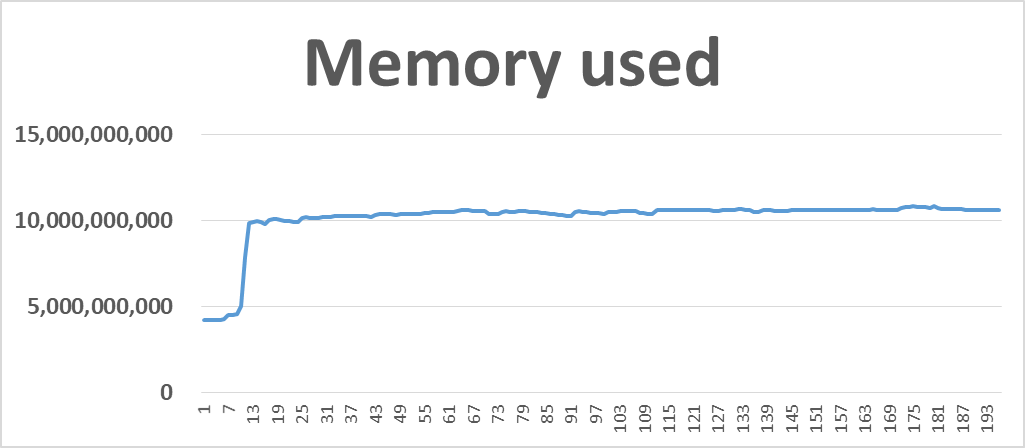

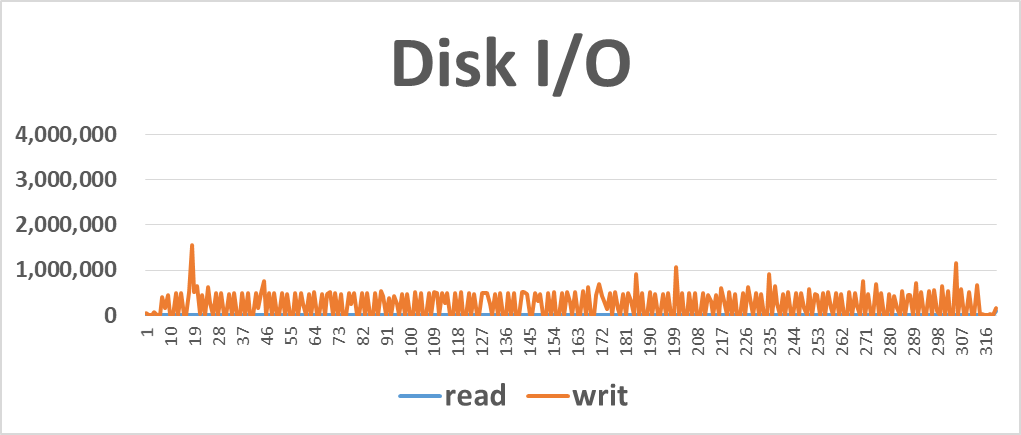

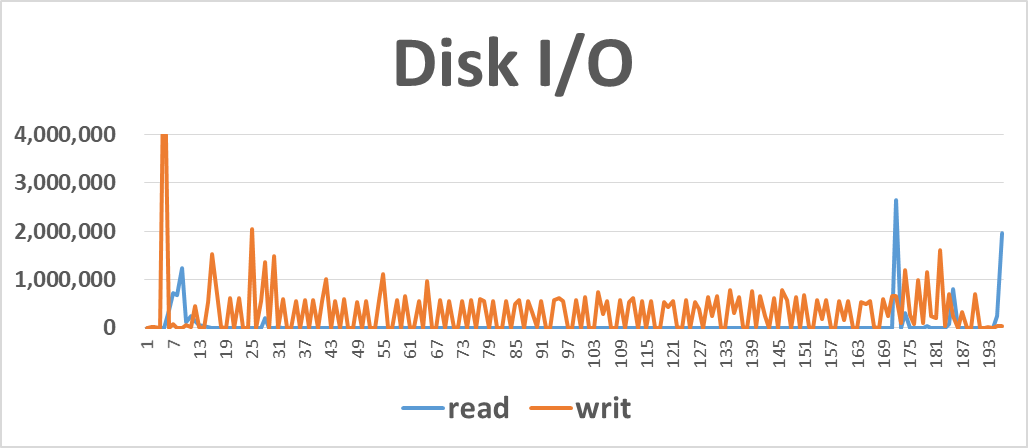

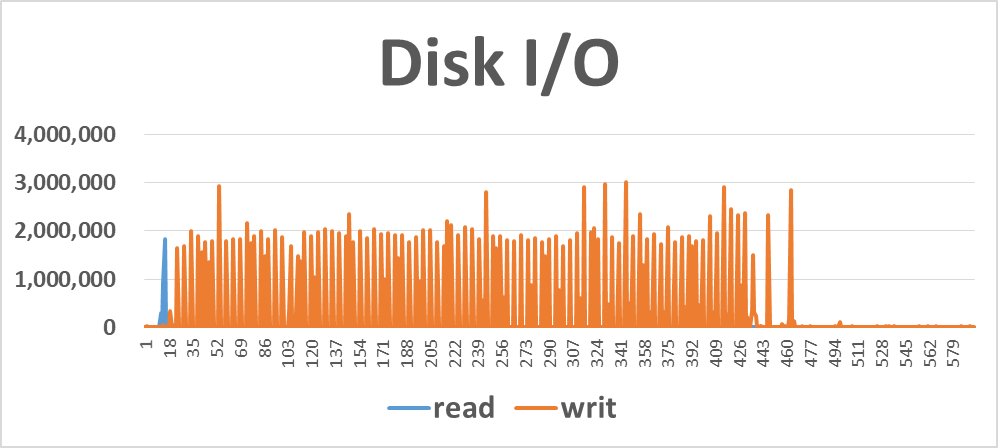

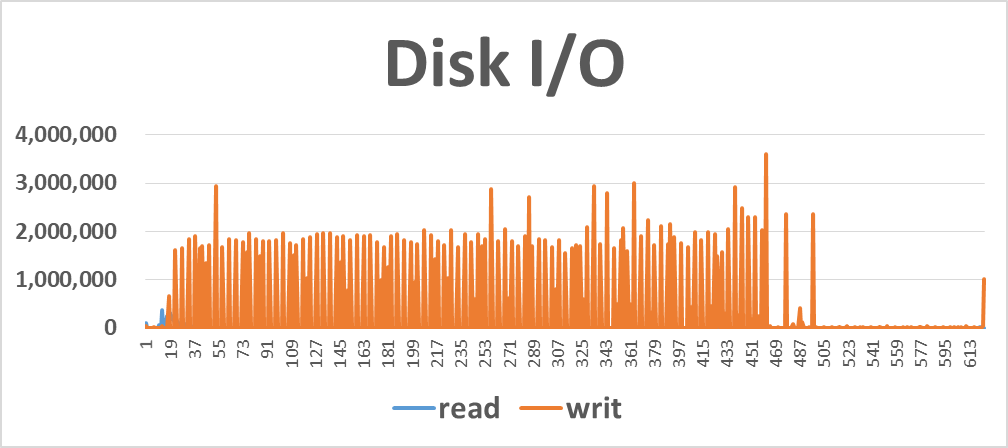

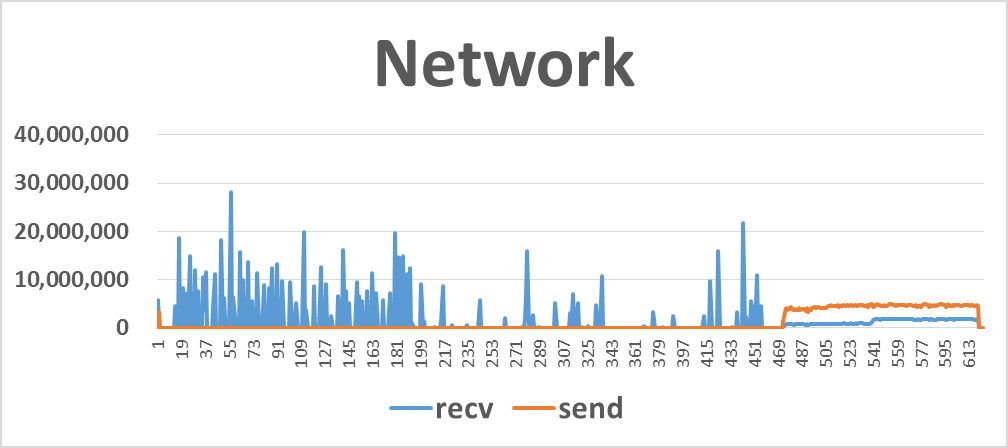

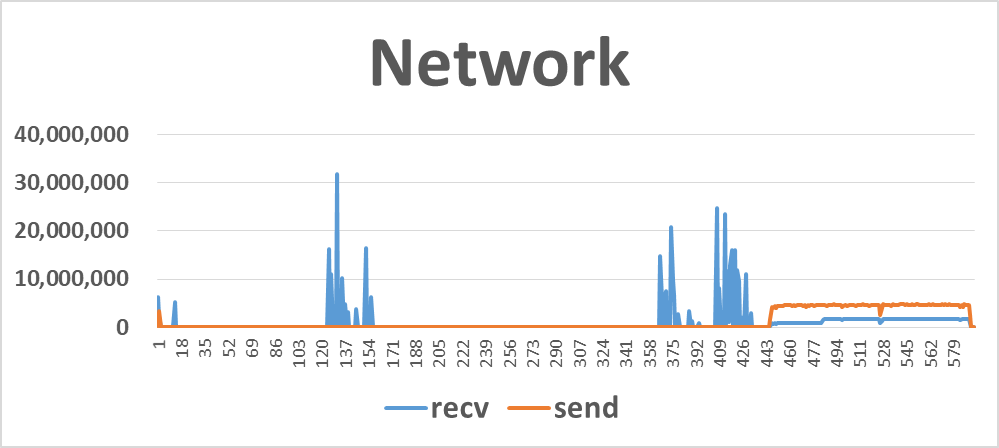

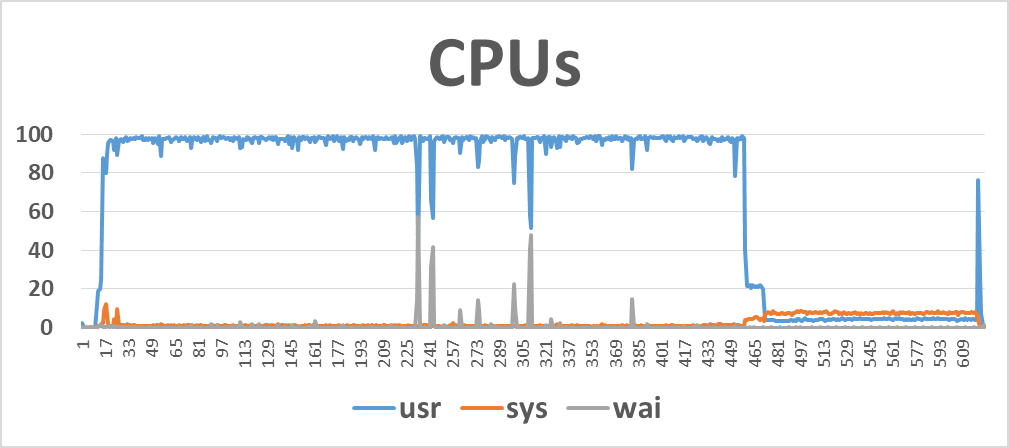

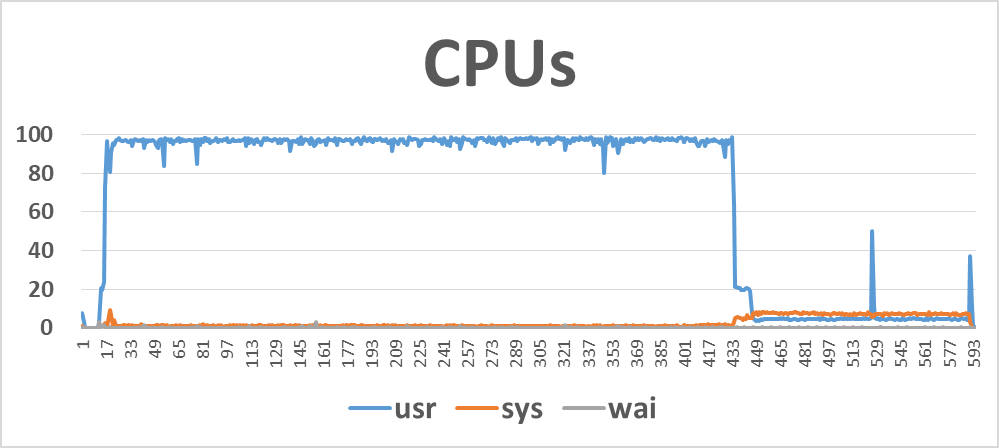

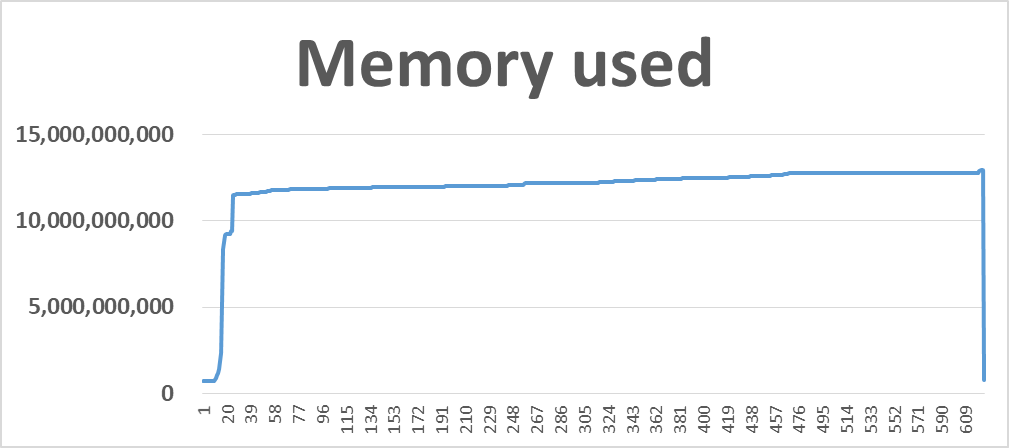

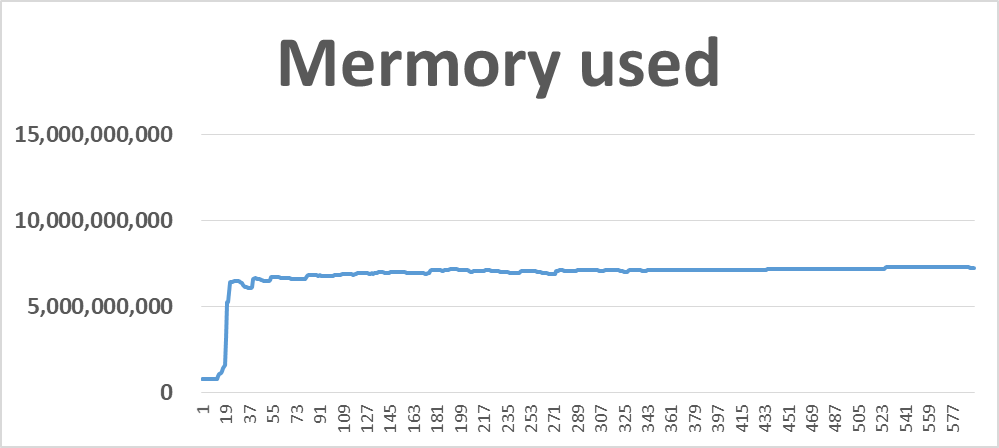


**Case4-8Core with 64GB memory**

**GATK-Indel Realigner (Step 7)**

**LOCAL DISK**

**MAHA**

**Case 3: 8Cores/30G memory**

**Case 4: 8Cores/ 64GB memory**

**Case 2: 8Cores/64GB memory**

**Case 1: 4cores/20GB memory**


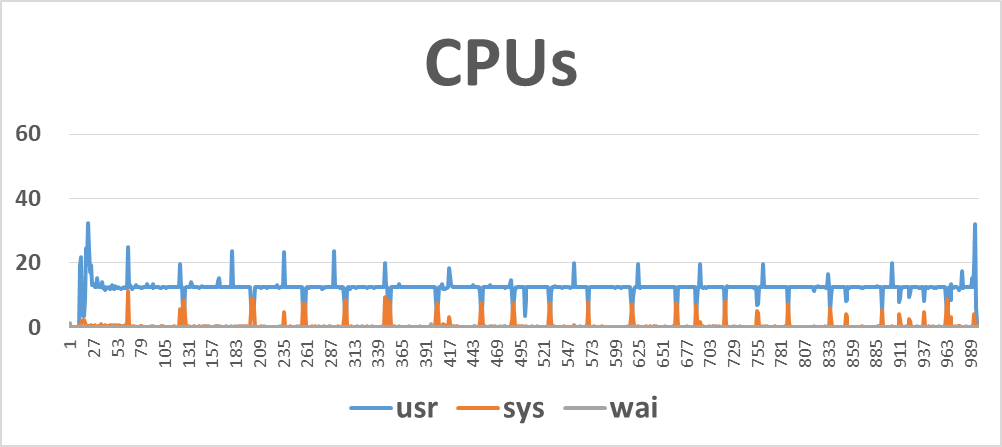

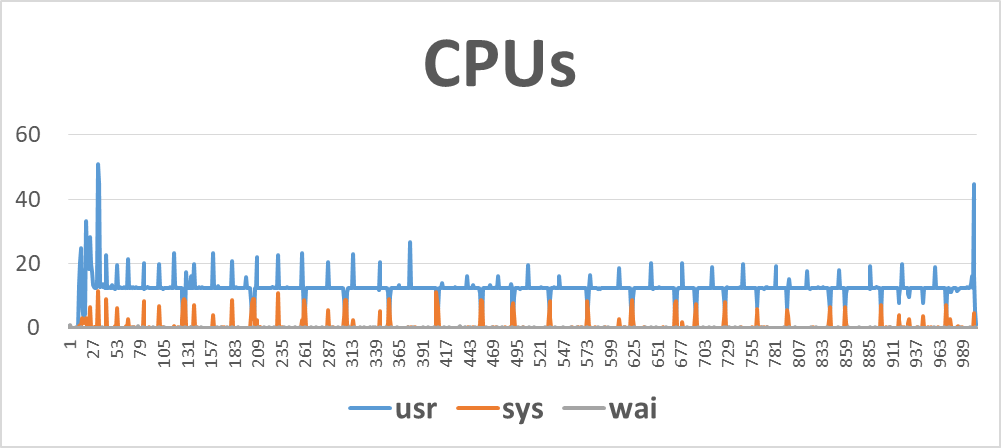

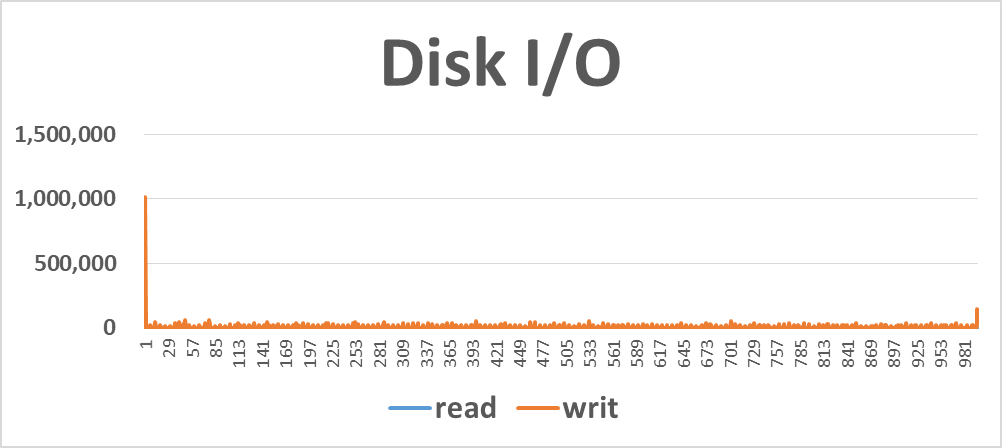

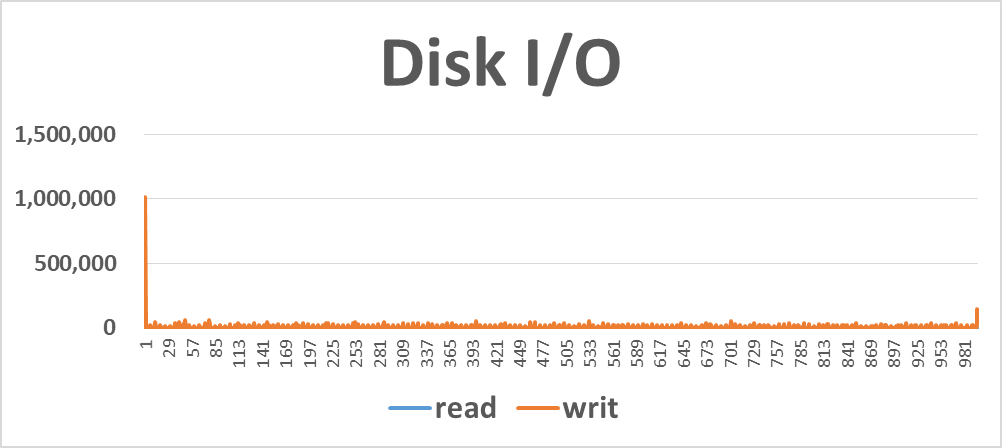

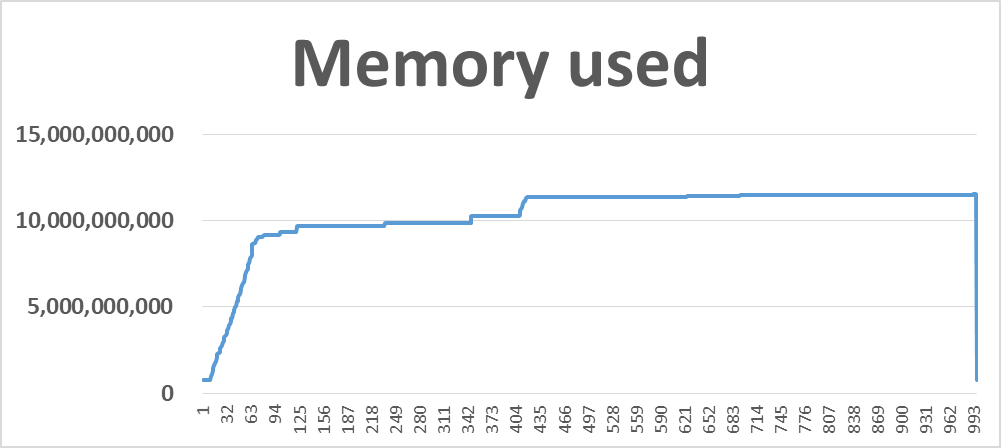

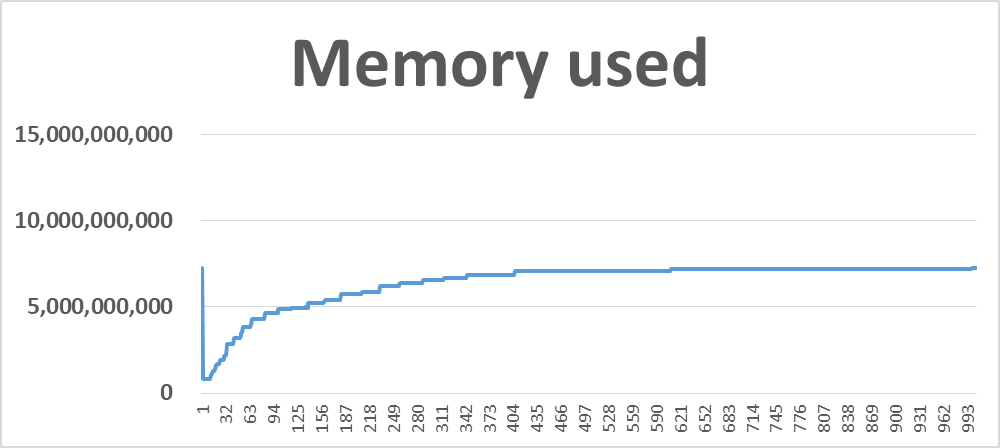

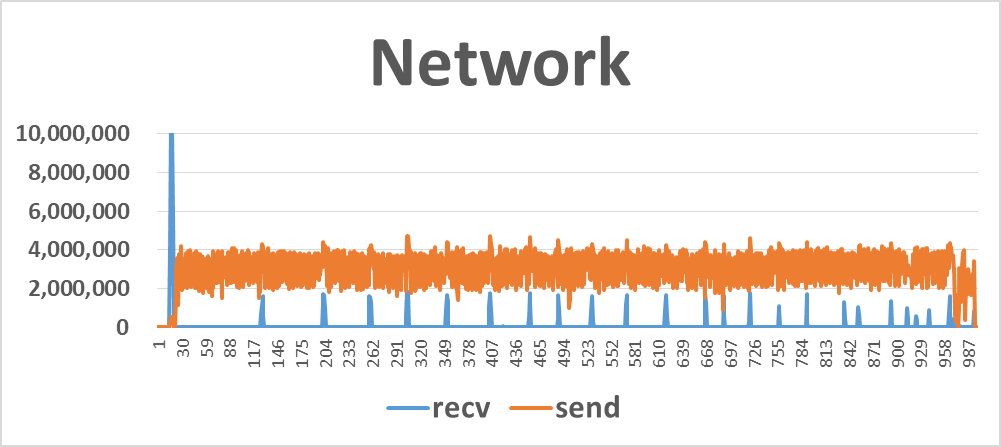

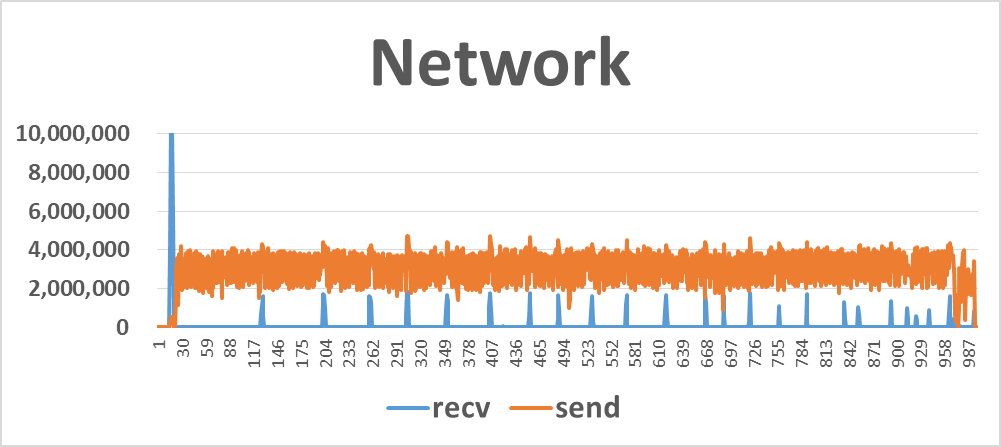

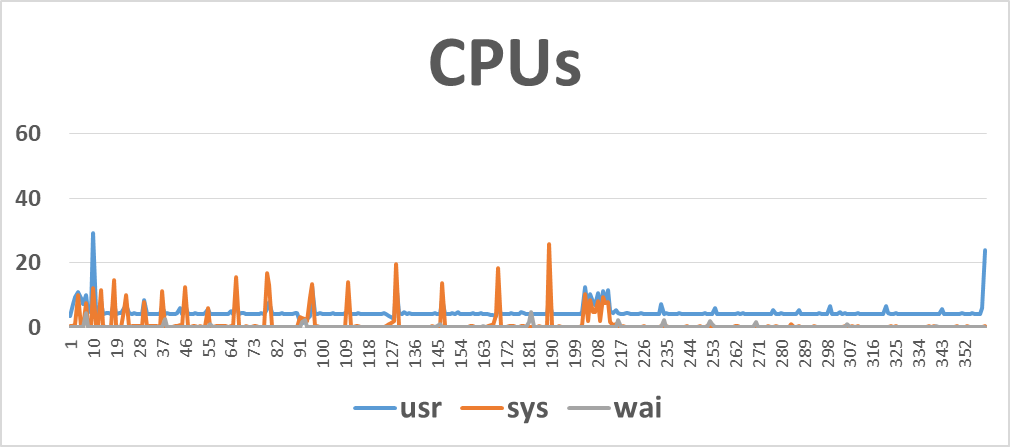

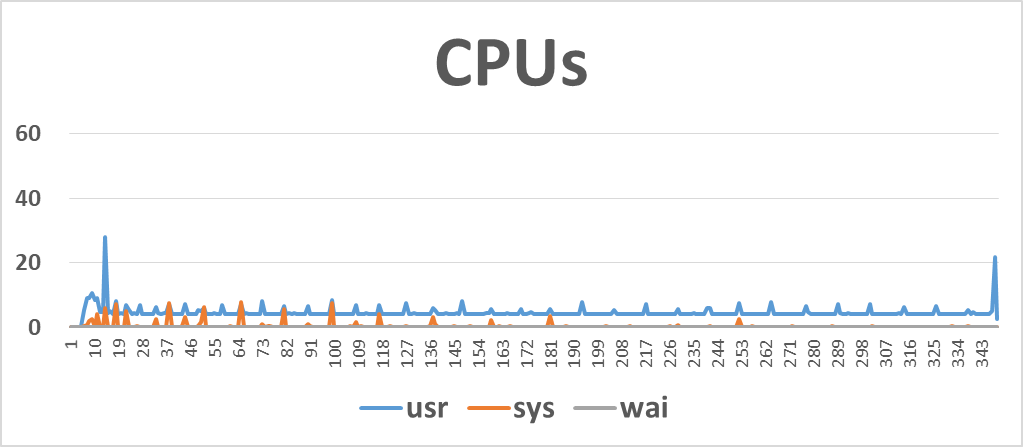

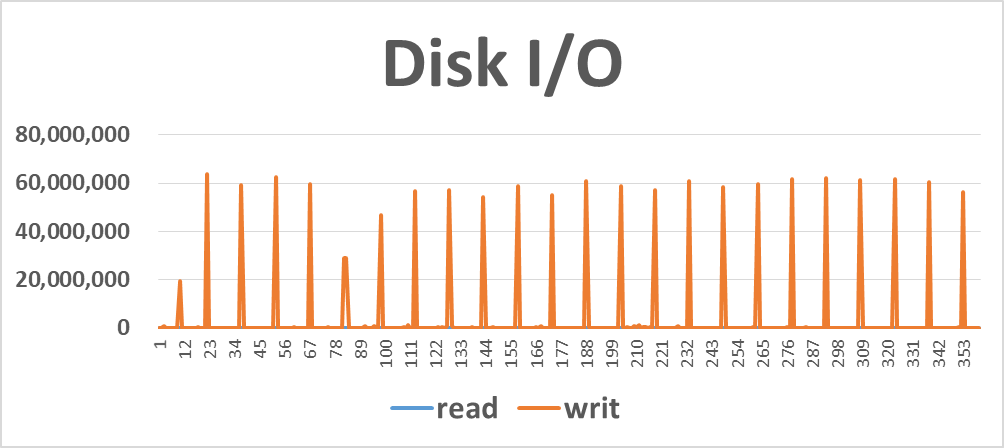

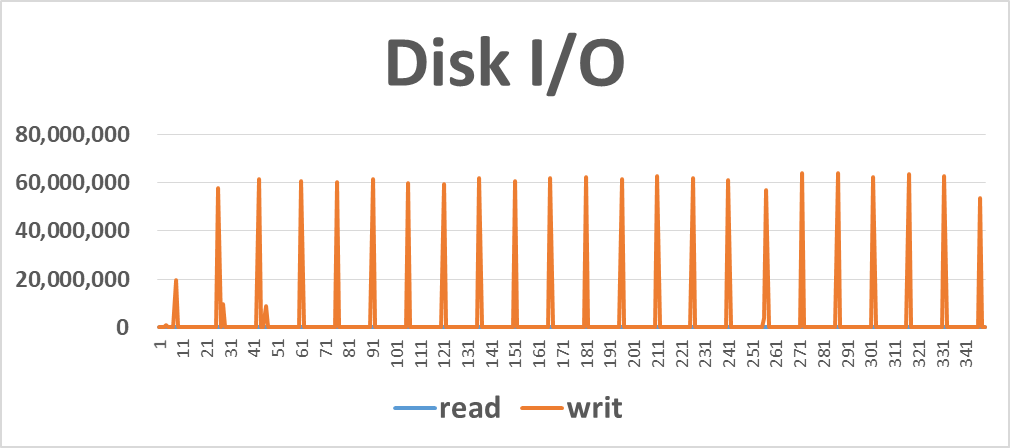

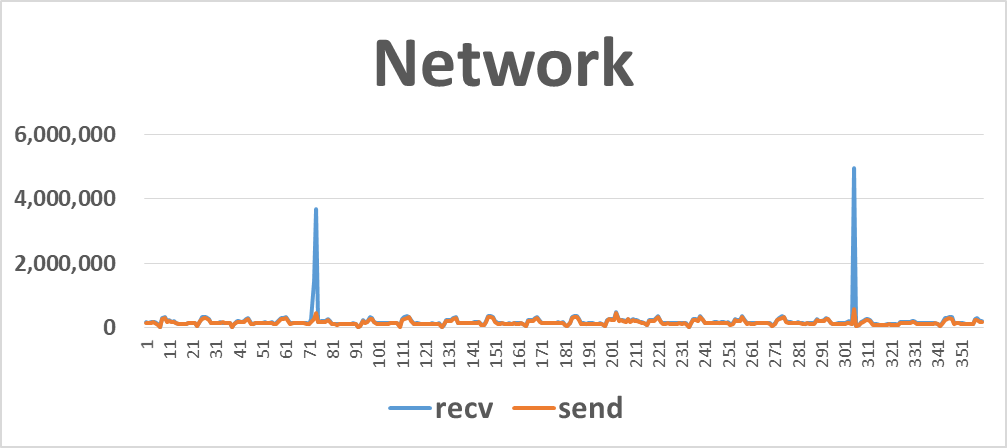

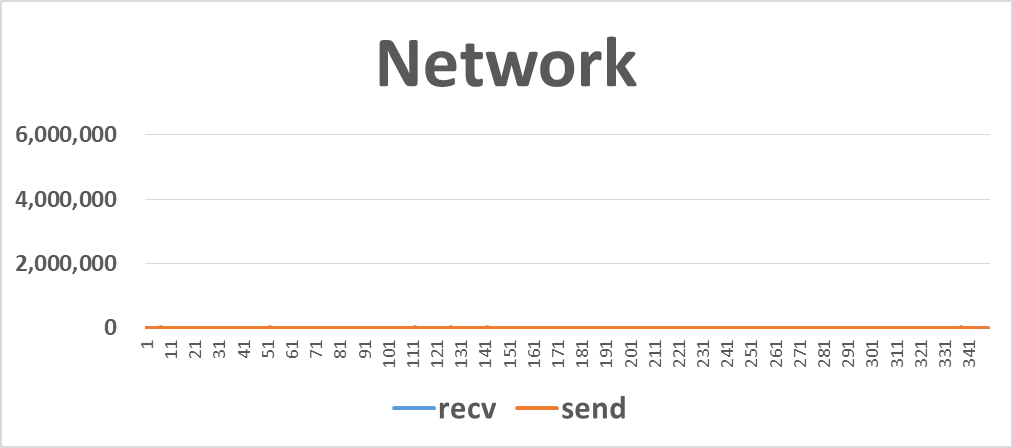

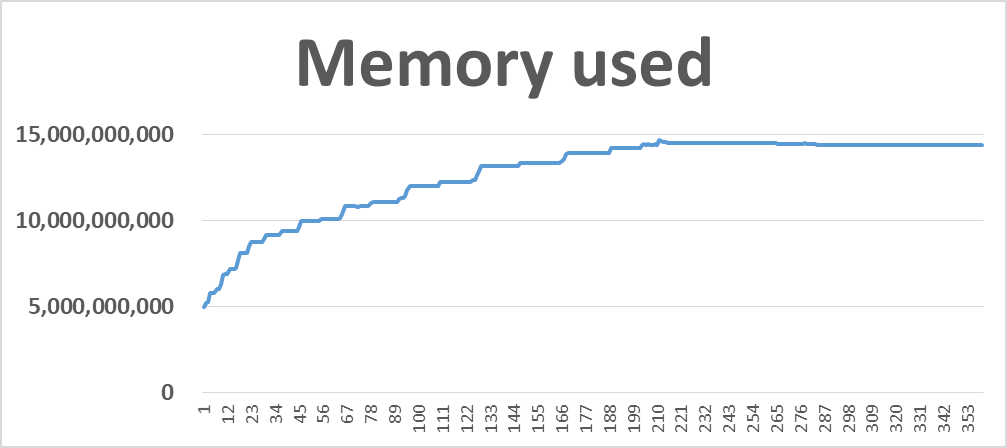

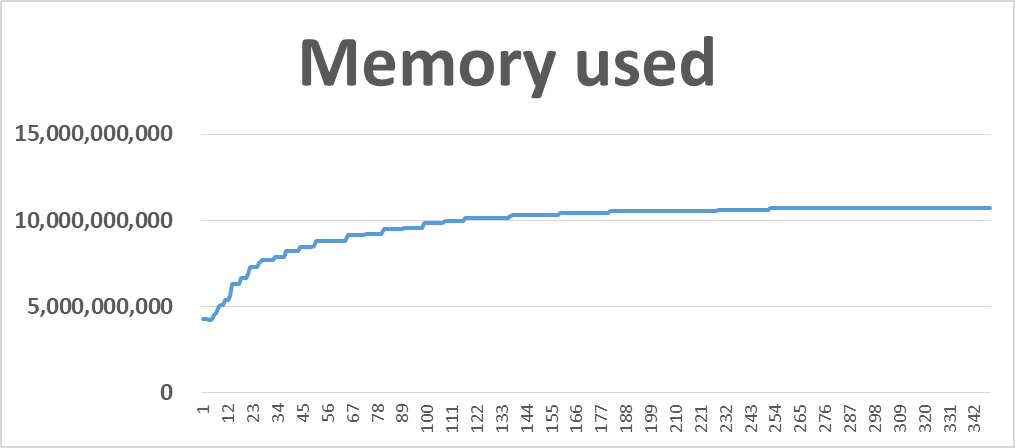


**GATK-Base Recalibrator (Step 8)**

**LOCAL DISK**

**MAHA**

**Case 1: 4cores/20GB memory**

**Case 2: 8Cores/64GB memory**

**Case 3: 8Cores/30G memory**

**Case 4: 8Cores/ 64GB memory**


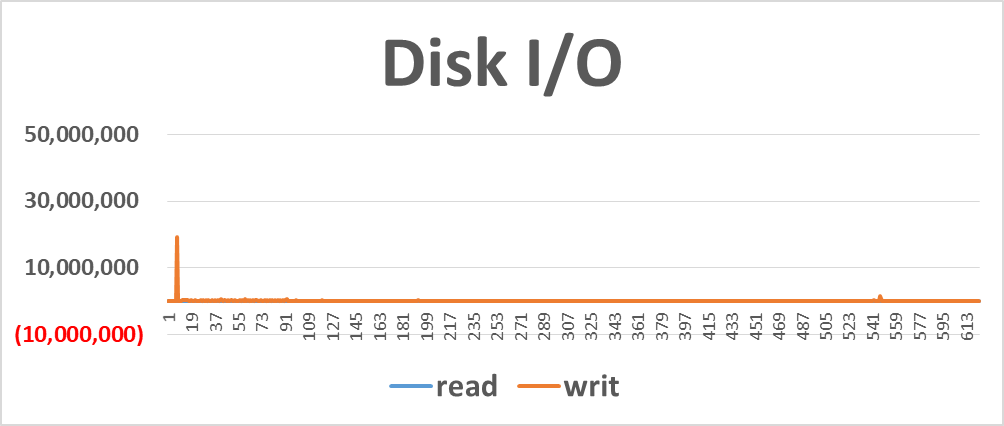

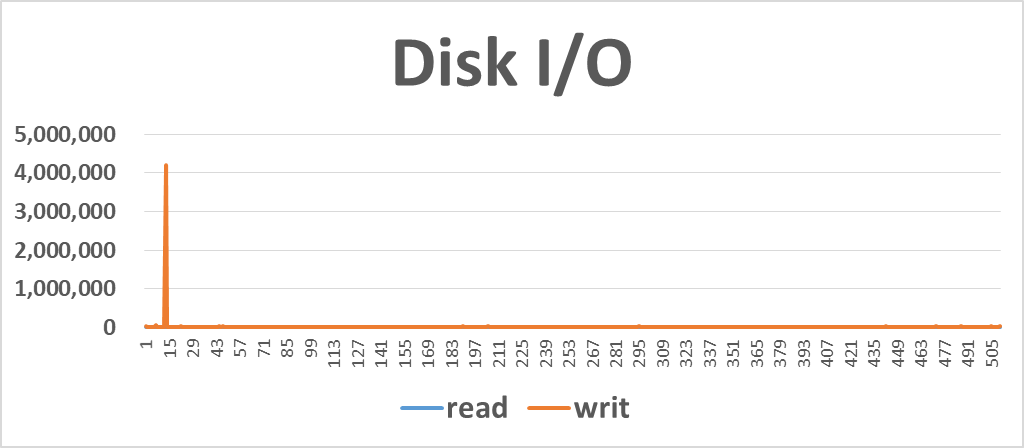

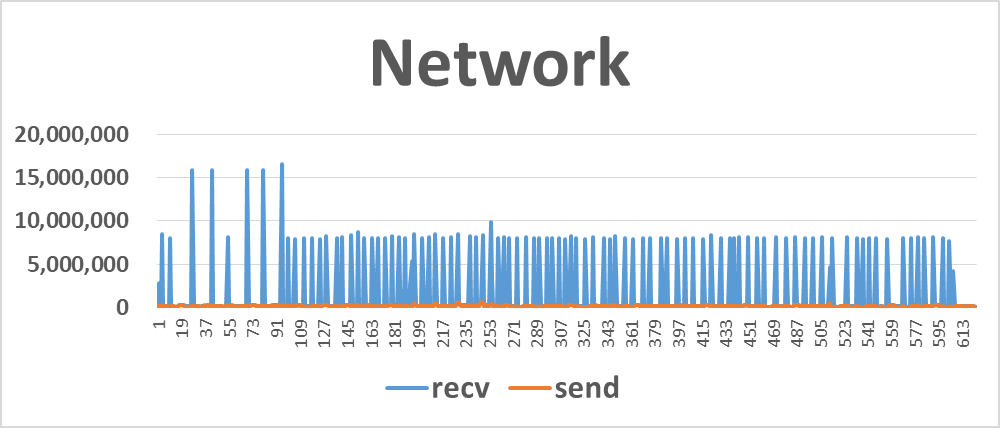

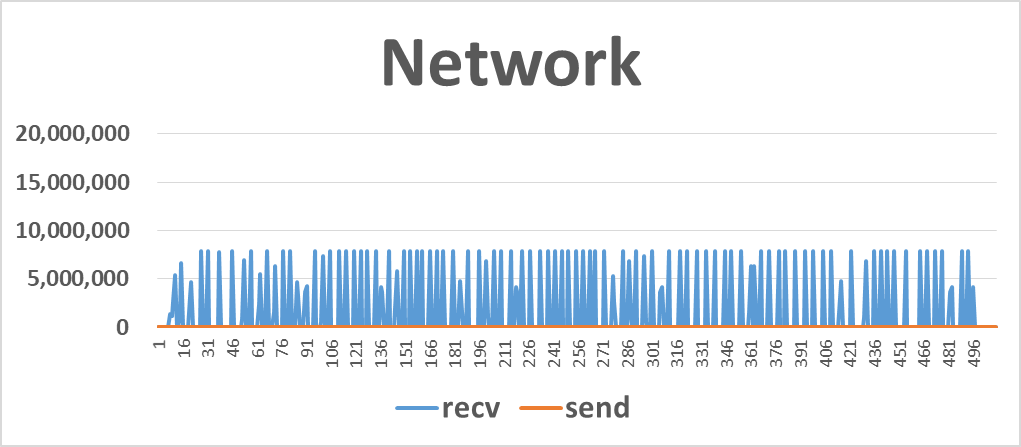


**GATK-Print Reads (Step 9)**

**MAHA**

**LOCAL DISK**

**Case 2: 8Cores/64GB memory**

**Case 3: 8Cores/30G memory**

**Case 4: 8Cores/ 64GB memory**

**Case 1: 4cores/20GB memory**

**GATK-Haplotype Caller (Step 10**

**MAHA**

**LOCAL DISK**

**Case 3: 8Cores/30G memory**

**Case 1: 4cores/20GB memory**

**Case 2: 8Cores/64GB memory**

**Case 4: 8Cores/ 64GB memory**
